# StableDNAm: towards a stable and efficient model for predicting DNA methylation based on adaptive feature correction learning

**DOI:** 10.1186/s12864-023-09802-7

**Published:** 2023-12-05

**Authors:** Linlin Zhuo, Rui Wang, Xiangzheng Fu, Xiaojun Yao

**Affiliations:** 1 College of Data Science and Artificial Intelligence, Wenzhou University of Technology, Wenzhou, 325000 China; 2 College of Computer Science and Electronic Engineering, Hunan University, Changsha, 410000 China; 3 Faculty of Applied Sciences, Macao Polytechnic University, Macao, 999078 China

**Keywords:** DNA methylation, Multi-scale and low-span features, Contrastive learning, Feature correction, Stability and scalability

## Abstract

**Background:**

DNA methylation, instrumental in numerous life processes, underscores the paramount importance of its accurate prediction. Recent studies suggest that deep learning, due to its capacity to extract profound insights, provides a more precise DNA methylation prediction. However, issues related to the stability and generalization performance of these models persist.

**Results:**

In this study, we introduce an efficient and stable DNA methylation prediction model. This model incorporates a feature fusion approach, adaptive feature correction technology, and a contrastive learning strategy. The proposed model presents several advantages. First, DNA sequences are encoded at four levels to comprehensively capture intricate information across multi-scale and low-span features. Second, we design a sequence-specific feature correction module that adaptively adjusts the weights of sequence features. This improvement enhances the model’s stability and scalability, or its generality. Third, our contrastive learning strategy mitigates the instability issues resulting from sparse data. To validate our model, we conducted multiple sets of experiments on commonly used datasets, demonstrating the model’s robustness and stability. Simultaneously, we amalgamate various datasets into a single, unified dataset. The experimental outcomes from this combined dataset substantiate the model’s robust adaptability.

**Conclusions:**

Our research findings affirm that the StableDNAm model is a general, stable, and effective instrument for DNA methylation prediction. It holds substantial promise for providing invaluable assistance in future methylation-related research and analyses.

**Supplementary Information:**

The online version contains supplementary material available at 10.1186/s12864-023-09802-7.

## Background

DNA methylation, a process involving the addition of methyl or hydroxymethyl groups to genetic molecules, is pivotal to significant biological reactions [1]. This modification of genetic molecules [2] regulates transcription [3, 4] and gene expression [5], playing an essential role in various life processes. For instance, it is crucial for the growth and development of certain mammalian species, as it orchestrates the silencing of specific gene categories, fosters the differentiation process of embryonic stem cells (ESCs), and upholds the stability of the parental genome [6]. Additionally, DNA methylation fluctuates throughout different life stages due to environmental factors. On the one hand, they can result in transcriptional inactivation and aging in living organisms [6, 7]. On the other hand, they may trigger diseases such as cancer [8–11].

Current research in DNA methylation primarily focuses on three types of modifications: 4mC (N4-methylcytosine), 5-Hydroxymethylcytosine (5hmC), and N6-methylenenene adenine (6mA) [12]. 4mC refers to the modification of the amino group at the fourth position of N-4 Cytosine, catalyzed by specific enzymes [13]. This modification can protect DNA from enzymatic degradation [14], playing a crucial role in DNA self-recognition, the regulation of DNA replication, and the correction of DNA replication errors [15]. 5hmC is the hydroxylated form of 5-methylcytosine (5mC), which can regulate genes and potentially be involved in cancer development [16]. Studies suggest that 5hmC can serve as a biomarker due to its diverse decoration patterns across different biological tissues [17]. Furthermore, 5hmC is involved in gene silencing, promoting the diverse functions of ESCs, tumorigenesis, among other processes [17]. 6mA refers to the methylation of adenine at the sixth position, facilitated by enzymes. It is a common, low-level modification method [18, 19] with functions that vary among different organisms [20]. For instance, it can uphold the stability of base pairing [21], regulate gene transcription, and play a pivotal defensive role in the host body. With the ongoing deterioration in the current ecological environment, which significantly impacts the regulation and expression processes of biosphere genes, identifying DNA methylation sites has become an urgent and vital task for exploring life development and preventing diseases.

In the early stages, DNA methylation detection primarily relied on traditional experimental techniques. Lister et al. utilized whole-genome sulfurous acid sequencing methods to analyze methylated Cytosine in human embryonic stem cells (ESCs) [22]. Meissner et al. proposed a method to decrease the randomness of sulfurous acid sequencing, thereby enabling the analysis of methylation decoration patterns [23]. Building on this, several innovative experimental methods have recently emerged. Hu et al. introduced a mapping technique known as “Jump seq” for 5hmC, which more effectively identifies and expands 5hmC [24]. Xiao et al. discovered that 6mA exists not only in prokaryotes but also to a lesser extent in human genes [9]. Ye et al. combined long-read technology to identify 4mC in Casuarina equisetifolia [25]. Despite these advancements, sequencing technologies still exhibit several drawbacks, such as low localization efficiency. Given that the three prevalent types of methylation often exist in a low-level form in nature, highly sensitive experimental methods are required for detection. As such, the development of computational methods for methylation prediction has become a pressing need to enhance efficiency and reduce costs.

Machine learning methods have seen substantial success in the field of biology [26–28], spurring the rapid advancement of research related to DNA methylation prediction. DNA4mC-LIP employs a linear weighting strategy to integrate six prediction models and constructs mixed features to predict 4mC sites [29]. DeepTorrent combines CNN and LSTM technologies to predict 4mC sites across multiple datasets, employing various encoders to enhance sequence representation, and utilizing Bayesian methods for model optimization [30]. Cong et al. introduced the MM-6mAPred model, grounded on the Markov model, to identify 6mA sites in DNA sequences [31]. Yu et al. suggested the SNNRice6mA model for predicting 6mA sites in rice, which stacks multiple one-dimensional CNN layers and fully connected layers. This model utilizes one-hot encoding for feature extraction as an alternative to traditional manual feature extraction [32]. The Deep6mA model, similar in techniques to the DeepTorrent model, predicts 6mA sites in rice and can also be applied to predict 6mA sites in other plants [33]. Building on this, Sho et al. proposed the BERT6mA model to predict 6mA, which integrates the GRU module and the Transformer encoder module, demonstrating robust performance [34]. Zhang et al. developed the iPromoter-5mC model, based on fully connected networks, to predict 5mC sites, incorporating three encoding features [35]. Additionally, BiLSTM-5mC trains multiple submodels based on Bi-LSTM and fully connected layers and executes integration operations to predict 5mC sites [36]. While these computational methods excel at predicting individual DNA methylation sites, they rarely consider or can be extended to the identification of multiple types of DNA methylation.

The key to solving the recognition task of multiple DNA methylation types lies in the adoption of appropriate feature encoding methods and universal models. Lv et al. integrated three different feature encoding methods and employed a random forest ensemble method to distinguish and identify various DNA methylation types, which proved to be effective [37]. Due to the need for improved feature extraction capabilities, Yu et al. subsequently designed a loss function based on conditional entropy techniques, utilizing self-attention mechanisms for adaptive feature encoding, thereby enhancing the recognition efficiency of different DNA methylation types [38]. In addition, Jin et al. achieved the current best performance in DNA methylation recognition by utilizing dual-scale encoding and a novel feature fusion approach [39]. For these state-of-the-art methods, multi-scale information is not fully extracted. There is no adaptive adjustment of weights for the extracted features, which may result in inability to apply to different datasets. In addition, the problem of sparse data is not considered.

Computational methods have been continuously refined, significantly enhancing the efficiency of DNA methylation predictions and expanding their applications. Despite these advancements, three main limitations persist. Firstly, there is the challenge of acquiring more robust feature representations. Secondly, constructing a stable and scalable model remains a significant obstacle. Lastly, dealing with sparse datasets remains an ongoing issue. To address these challenges, we propose a model based on adaptive feature correction learning, named StableDNAm. First, our model integrates multi-scale and low-span features to enhance the robustness of sequence representations. Second, we design a sequence-based feature correction module that adaptively adjusts feature weights by strengthening local features, contributing to the model’s stability and scalability. Finally, we apply a contrastive learning module that enhances the model’s stability and adaptability, particularly when dealing with sparse datasets. Our main contributions are as follows:

1. Leveraging the transformer encoder, we develop a DNA methylation recognition model, termed StableDNAm, which has demonstrated trustworthy performance. Additionally, we consolidated the training and testing sets from seventeen datasets, which further attests to the scalability of the StableDNAm model in this unified dataset.
2. We craft a low-span, multi-scale feature fusion methodology that integrates four-scale features of $3-mer, 4-mer, 5-mer$, and $6-mer$ DNA sequences. This design allows for the extraction of more intricate information while ensuring important data across different datasets is not lost.
3. We engineer a feature correction module named 2-D SENET, capable of adaptively adjusting the weight of sequence features for diverse datasets. This allows our model to accommodate diverse types of methylation datasets, thereby substantially enhancing the model’s stability and scalability.
4. We incorporate a contrastive learning module into the model’s training process, thereby enhancing the model’s adaptability when dealing with sparse datasets.

## Results

### Datasets

We primarily evaluate StableDNAm and its comparative models using two sets of datasets. The first set comprises seventeen standard DNA methylation datasets as described in prior research [39]. These datasets include 5hmC_H.sapiens, 5hmC_M.musculus, 4mC_C.equisetifolia, 4mC_F.vesca, 4mC_S.cerevisiae, 4mC_Tolypocladium, 6mA_A.thaliana, 6mA_C.elegans, 6mA_C.equisetifolia, 6mA_D.melanogaster, 6mA_F.vesca, 6mA_H.sapiens, 6mA_R.chinensis, 6mA_S.cerevisiae, 6mA_T.thermophile, 6mA_Tolypocladium, and 6mA_XocBLS256. For the sake of brevity, we denote these as D1 through D17, respectively. The second set forms a comprehensive dataset, which is a combination of the aforementioned seventeen methylation datasets, subsequently split into a 1:1 ratio into training and testing subsets. To demonstrate our data distribution, we compiled the distribution of positive and negative samples across all datasets, as shown in Table 1. It is evident that the sample sizes vary across different datasets, with some datasets containing tens of thousands of samples while others have only a few hundred.

**Table 1:** The number of positive and negative samples in the datasets

Datasets	Positive samples	Negative samples
5hmC_H.sapiens	2344	2344
5hmC_M.musculus	3680	3680
4mC_C.equisetifolia	366	366
4mC_F.vesca	15796	15796
4mC_S.cerevisiae	2000	2000
4mC_Tolypocladium	15326	15326
6mA_A.thaliana	31872	31872
6mA_C.elegans	7960	7960
6mA_C.equisetifolia	6066	6066
6mA_D.melanogaster	11190	11190
6mA_F.vesca	3100	3100
6mA_H.sapiens	18334	18334
6mA_R.chinensis	598	598
6mA_S.cerevisiae	3786	3786
6mA_T.thermophile	107600	107600
6mA_Tolypocladium	3378	3378
6mA_Xoc BLS256	17214	17214
Unified dataset	267824	267824

### Evaluation metrics

To evaluate the performance of the StableDNAm model relative to other comparative models, we employed common performance metrics in this study, including Accuracy (ACC), Sensitivity (SN), Specificity (SP), and Matthew’s Correlation Coefficient (MCC). The computation of these metrics is as follows:

1 $$\begin{aligned} ACC=\frac{TP+TN}{TP+TN+FP+FN}, \end{aligned}$$

2 $$\begin{aligned} SN=\frac{TP}{TP+FN}, \end{aligned}$$

3 $$\begin{aligned} SP=\frac{TN}{TN+FP}, \end{aligned}$$

4 $$\begin{aligned} MCC=\frac{TP*TN-FP*FN}{\sqrt{(TP+FP)(TP+FN)(TN+FP)(TN+FN)}}, \end{aligned}$$

5 $$\begin{aligned} PRE = \frac{TP}{TP + FP}, \end{aligned}$$

6 $$\begin{aligned} F1 = \frac{2 * \text {PRE} * \text {SN}}{\text {PRE} + \text {SN}}, \end{aligned}$$

where *TP* indicates the number of DNA methylated correctly identified, *FN* indicates the number of DNA methylated as unmethylated, *TN* indicates the number of unmethylated correctly identified, and *FP* indicates the number of DNA unmethylated identified is the number of methylations. *ACC* and *MCC* are comprehensive indicators for performance evaluation. In addition, this study also utilizes AUROC and AUPR curves [40] to evaluate the overall performance of the model. In this study, AUROC can be commonly abbreviated as *AUC* (the area under the ROC curve). These curves provide an intuitive visualization of the model’s superiority.

### Comparison with other models

**Fig. 1:**
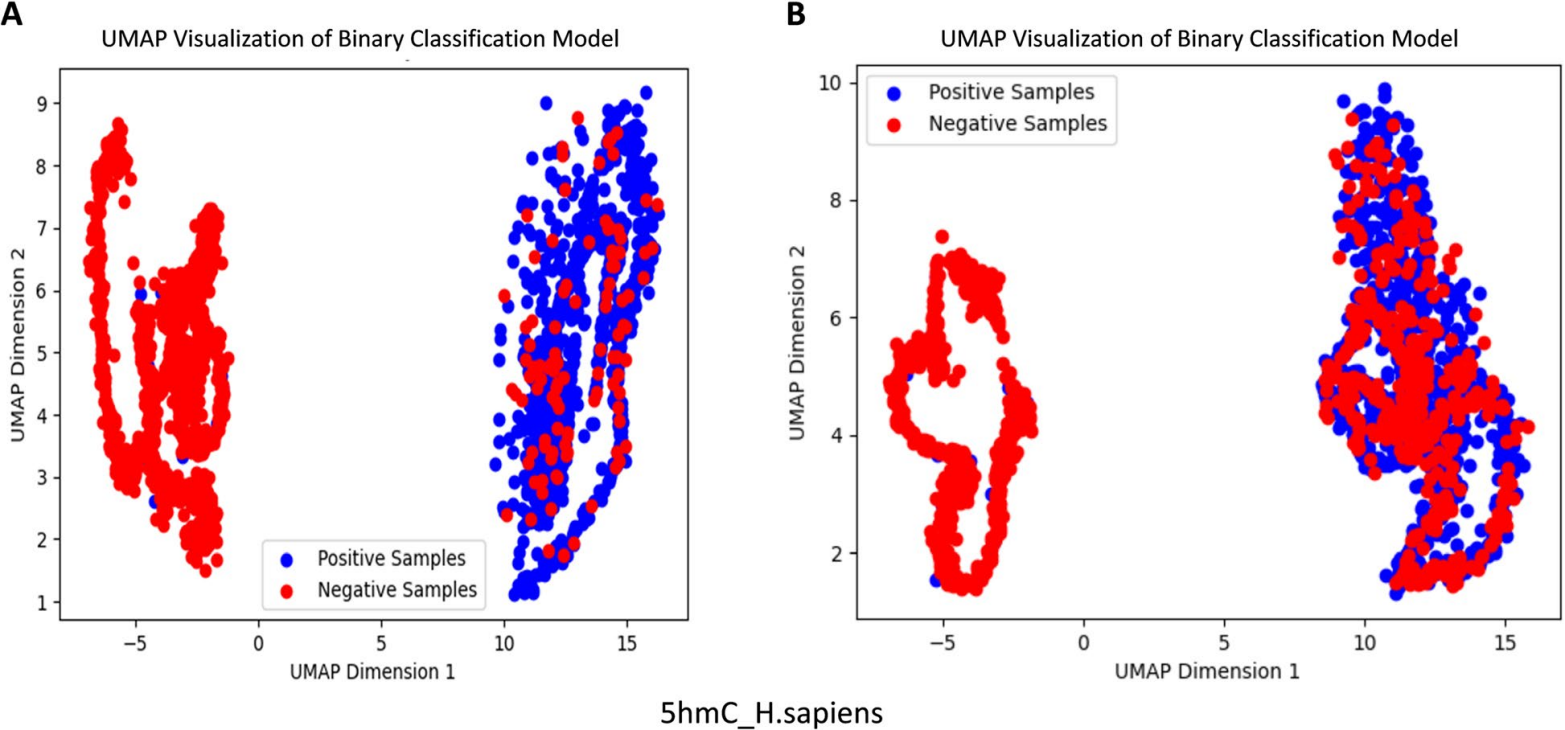
Comparison of UMAP visualization results of the model on 5hmC_H.sapiens dataset

We compared the proposed StableDNAm model with six current standard models across seventeen distinct datasets. Table 2 presents the *ACC*, *AUC*,  and *MCC* metrics for all models on these datasets, respectively. Performance comparisons using other metrics can be found in the Supplementary file (All-indicators.xlsx). Details on the training parameters for the other models (training_parameters.pdf) and the standard deviations for each dataset (k-mer.xlsx) are also available in the Supplementary files. Evidently, our model outperforms the existing six models in 12 out of the 17 datasets. Specifically, the average *ACC* index of the proposed StableDNAm model exceeds the second and third ranked models, iDNA-ABF and iDNA-ABT, by 1.6% and 0.87%, average AUC by 2.17% and 1.33%, and average MCC by 1.88% and 3.10% respectively. Importantly, on the datasets 4mC_C.equisetifolia, 6mC_C.elegans, and 6mA_C.equisetifolia, the StableDNAm model showcases significant improvements, with the *ACC* scores increasing by ranges of 2.8%-14.22%, 1.83%-18.63%, and 1.17%-7.48% respectively. In addition to the *ACC* scores, similar trends can be observed in the *AUC* and *MCC* metrics. To Intuitively illustrate the superior performance of the proposed StableDNAm model compared to other methods, we utilize the Uniform Manifold Approximation and Projection (UMAP) [41] technique for visualizing the distribution within the model’s output feature representation space. UMAP is a commonly adopted visualization tool that discloses crucial data attributes through dimensionality reduction. For visual comparison, we selected the second-best performing model, iDNA-ABF, and analyzed it alongside the proposed StableDNAm model on the 5hmC_H.sapiens dataset. As illustrated in Fig. 1, Subfigures A and B display the feature space distribution of the StableDNAm model and the iDNA-ABF model on the 5hmC_H.sapiens dataset, respectively. Dots symbolize samples, with methylated sites (positive samples) marked in blue and unmethylated sites (negative samples) in red. As seen in Subfigure A, the proposed StableDNAm model distinctly segregates positive and negative samples, with each class clustering tightly without scattering. Meanwhile, as observed in Subfigure B, though the feature space of the iDNA-ABF model demarcates some boundary between positive and negative samples, the positive and negative samples in the right section are essentially fused, posing limitations for a classification model.

**Table 2:** Comparison of the performance of StableDNAm with six state of the art models on the seventeen datasets

Indicator	Models	D1	D2	D3	D4	D5	D6	D7	D8	D9	D10	D11	D12	D13	D14	D15	D16	D17
*ACC*	iDNA_MS [37]	0.948	0.968	0.711	0.824	0.704	0.712	0.838	0.856	0.711	0.896	0.923	0.884	**0.855**	0.786	0.856	0.734	0.845
	iDNA_ABT [38]	**0.949**	**0.969**	0.825	0.842	0.703	0.738	0.854	0.890	0.733	0.912	0.927	0.898	0.826	0.801	0.874	**0.774**	0.869
	iDNA_ABF [39]	**0.949**	0.966	0.814	0.851	0.680	0.687	0.859	0.864	0.729	0.917	0.938	0.902	0.789	0.826	0.880	0.730	**0.878**
	BERT6mA [34]	0.947	0.963	0.773	0.822	0.691	0.735	0.853	0.902	0.721	0.882	0.926	0.896	0.781	0.813	0.874	0.752	0.863
	Deep6mA [33]	0.932	0.960	0.760	0.850	0.687	0.737	**0.861**	0.902	0.729	0.920	0.925	0.899	0.816	0.801	0.872	0.762	0.842
	MM-6mAPred [31]	0.906	0.931	0.773	0.764	0.689	0.670	0.755	0.722	0.670	0.809	0.851	0.823	0.786	0.745	0.747	0.705	0.751
	**StableDNAm**	**0.949**	0.968	**0.853**	**0.853**	**0.710**	**0.743**	**0.861**	**0.909**	**0.745**	**0.923**	**0.939**	**0.907**	0.818	**0.827**	**0.882**	0.768	0.877
*AUC*	iDNA_MS [37]	0.962	0.984	0.780	0.900	0.761	0.780	0.909	0.935	0.785	0.956	0.975	0.950	**0.926**	0.868	0.926	0.821	0.925
	iDNA_ABT [38]	0.955	0.976	0.856	0.902	0.753	0.799	0.918	0.943	0.798	0.942	0.955	0.941	0.859	0.871	0.928	0.836	0.926
	iDNA_ABF [39]	**0.969**	**0.986**	0.878	**0.928**	0.728	0.748	0.932	0.936	0.811	0.960	0.979	0.964	0.889	0.903	0.941	0.804	0.945
	BERT6mA [34]	0.956	0.975	0.799	0.903	0.742	0.805	0.915	0.962	0.777	0.950	0.962	0.947	0.865	0.850	0.938	0.838	0.925
	Deep6mA [33]	0.953	0.974	0.886	0.926	0.759	0.814	0.933	0.945	0.798	0.968	0.972	0.964	0.888	0.884	**0.944**	0.814	**0.949**
	MM-6mAPred [31]	0.939	0.978	0.886	0.844	0.755	0.735	0.834	0.831	0.708	0.892	0.954	0.901	0.853	0.821	0.843	0.771	0.828
	**StableDNAm**	0.967	0.981	**0.896**	**0.928**	**0.776**	**0.819**	**0.934**	**0.966**	**0.816**	**0.971**	**0.981**	**0.969**	0.881	**0.905**	**0.944**	**0.845**	**0.949**
*MCC*	iDNA_MS [37]	0.897	0.935	0.422	0.648	0.408	0.423	0.676	0.712	0.423	0.792	0.846	0.769	0.710	0.572	0.728	0.468	0.691
	iDNA_ABT [38]	**0.901**	**0.937**	0.652	0.684	0.406	0.477	0.709	0.781	0.467	0.824	0.824	0.796	**0.653**	0.610	0.754	**0.551**	0.739
	iDNA_ABF [39]	0.900	0.933	0.635	0.704	0.361	0.373	0.719	0.728	0.460	0.833	0.877	0.805	0.586	**0.670**	0.768	0.460	**0.756**
	BERT6mA [34]	0.896	0.926	0.562	0.653	0.383	0.471	0.705	0.804	0.443	0.769	0.851	0.792	0.564	0.627	0.752	0.505	0.727
	Deep6mA [33]	0.864	0.920	0.547	0.701	0.375	0.475	**0.722**	0.803	0.458	0.840	0.850	0.797	0.633	0.602	0.755	0.525	0.742
	MM-6mAPred [31]	0.820	0.865	0.556	0.528	0.378	0.340	0.511	0.478	0.344	0.6202	0.720	0.646	0.573	0.491	0.596	0.427	0.506
	** StableDNAm**	0.900	0.936	**0.706**	**0.706**	**0.423**	**0.487**	**0.722**	**0.818**	**0.494**	**0.846**	**0.878**	**0.815**	0.635	0.654	**0.772**	0.538	**0.756**

Bold indicates the optimal value among the compared methods, and underline indicates the suboptimal value

The StableDNAm model demonstrates significantly higher discrimination between the two classes of samples in the feature space compared to the second-best performing iDNA-ABF model. This signifies that our model is proficient in learning robust feature representations from diverse class samples. This may be attributed to several factors. Firstly, the robust pre-training model aids in capturing more latent contextual semantic information from millions of background genome sequences. Secondly, the model is effectively fine-tuned based on the dataset. Moreover, the fusion of multiple features including $3-mer, 4-mer, 5-mer$, and $6-mer$, coupled with the use of SENET technology for adaptive feature adjustment on various methylation datasets, contributes to the robust performance of our model. Conversely, even though the iDNA-ABF model also utilizes pre-training, it appears to struggle with effective fine-tuning in downstream datasets. This issue might be related to overfitting, a problem we will further demonstrate in subsequent experiments.

### Comparison on a unified dataset

**Fig. 2:**
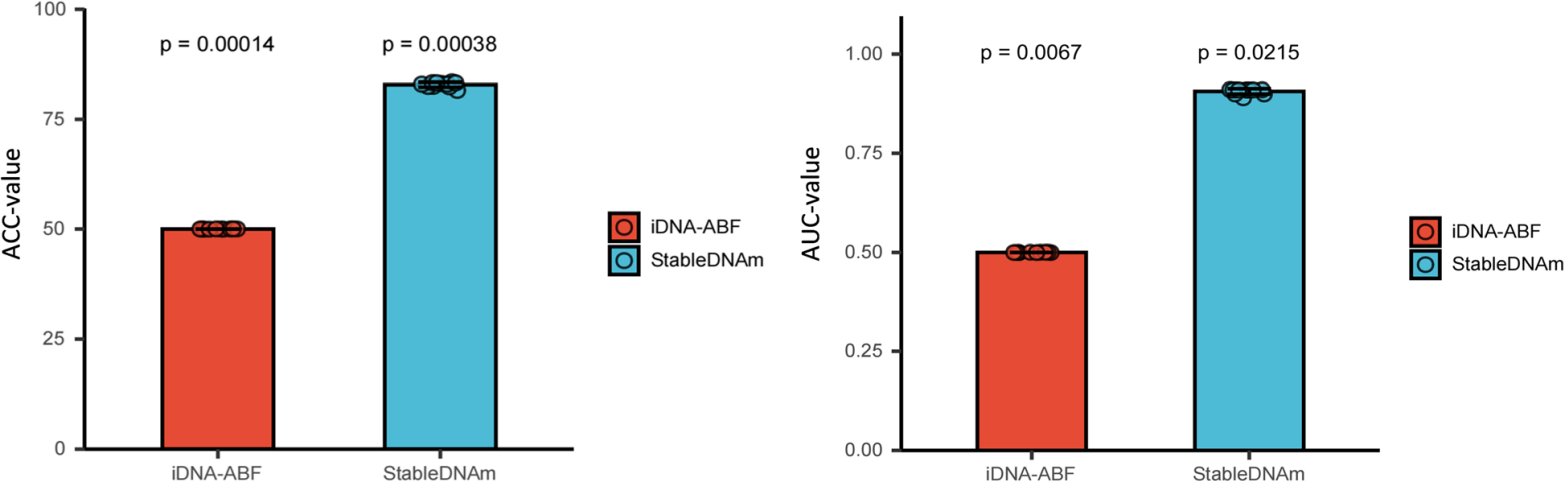
Performance of the iDNA-ABF model and the StableDNAm model on a unified dataset measured by ACC and AUC metrics

To evaluate the adaptability of our proposed StableDNAm model, we composed a unified dataset by amalgamating seventeen different species and multiple types of methylation datasets. We then divided this collective dataset into distinct training and testing sets. Concurrently, we conducted comparative tests with the iDNA-ABF model, which has proven to perform second-best across various datasets. As depicted in Fig. 2, the comparative performance of the StableDNAm and iDNA-ABF models on the unified dataset reveals distinct differences. The *ACC* and *MCC* of the iDNA-ABF model on the unified dataset, showcased on the left plot of Fig. 2, hover around the 50% mark, indicative of its insufficient predictive abilities. This performance alludes to the iDNA-ABF model’s specialization for individual methylation types, and its limited adaptability to a diverse range of methylation varieties. In contrast, the StableDNAm model demonstrates significant adaptability, achieving an *ACC* of 83.5% and an *AUC* of 91.0%. The p-value, derived from T-tests and displayed in the figure, further substantiates the model’s stability, suggesting its capacity to comprehend and generalize across an array of data types after training. Our proposed model leverages a unified dataset that assimilates a variety of methylation data types, thus empowering the model with extensive knowledge. Conversely, the iDNA-ABF model may be constricted to specific data types, resulting in limited scalability. This disparity might stem from the fact that our model can be effectively fine-tuned based on pre-training, while the iDNA-ABF model is excessively reliant on its pre-trained state, making downstream fine-tuning challenging. Following this analysis, it’s evident that the StableDNAm model can be both trained and utilized for predictions based on a consolidated methylation dataset, circumventing the need for individual training for different methylation data types. This indicates that the StableDNAm model serves as a general tool for DNA methylation prediction, significantly diminishing the intricacies of methylation analysis while enhancing its efficiency.

### Model stability and robustness

**Fig. 3:**
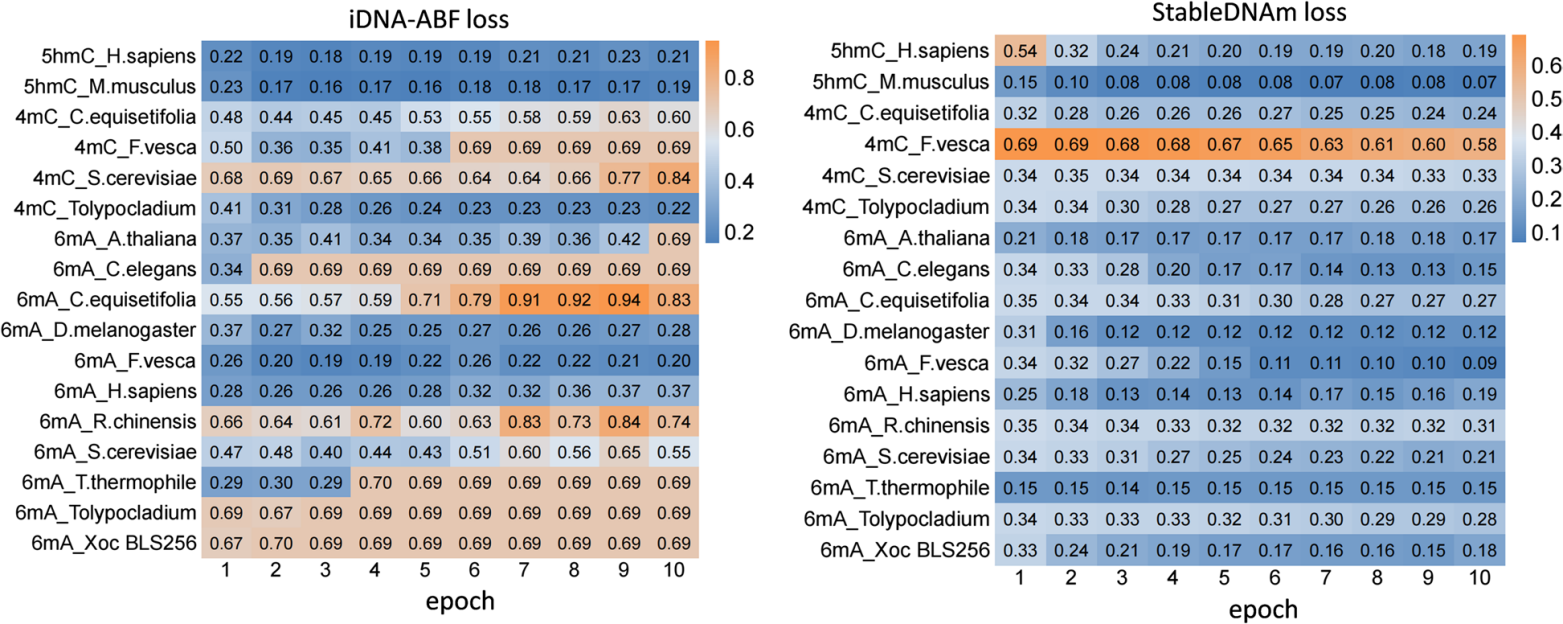
Performance of the iDNA-ABF model and the StableDNAm model on seventeen datasets concerning the loss during training

**Fig. 4:**
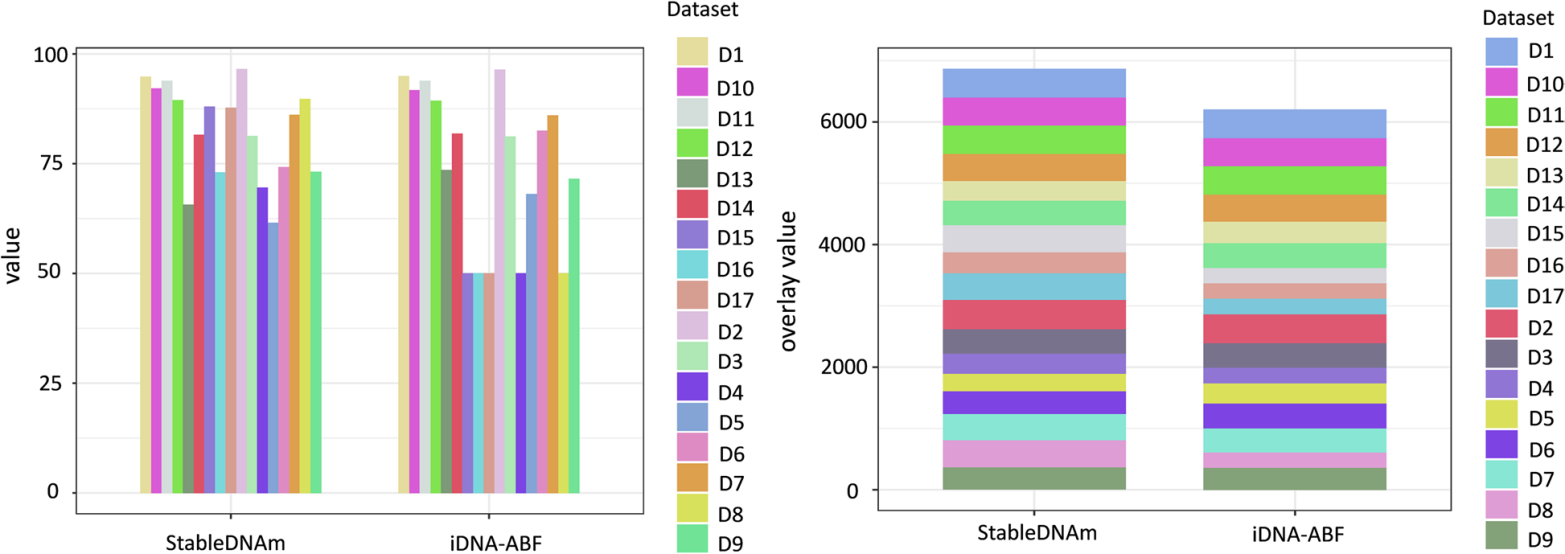
Performance of the iDNA-ABF model and the StableDNAm model on seventeen datasets with respect to the ACC over the last five epochs

**Fig. 5:**
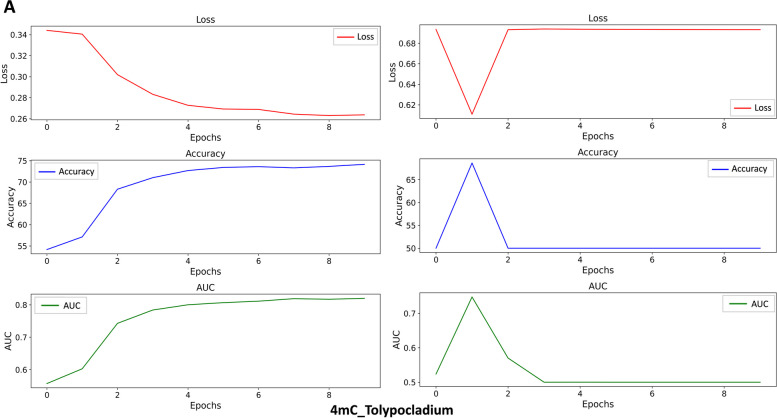
Performance comparison of the models on the 4mC_Tolypocladium dataset

**Fig. 6:**
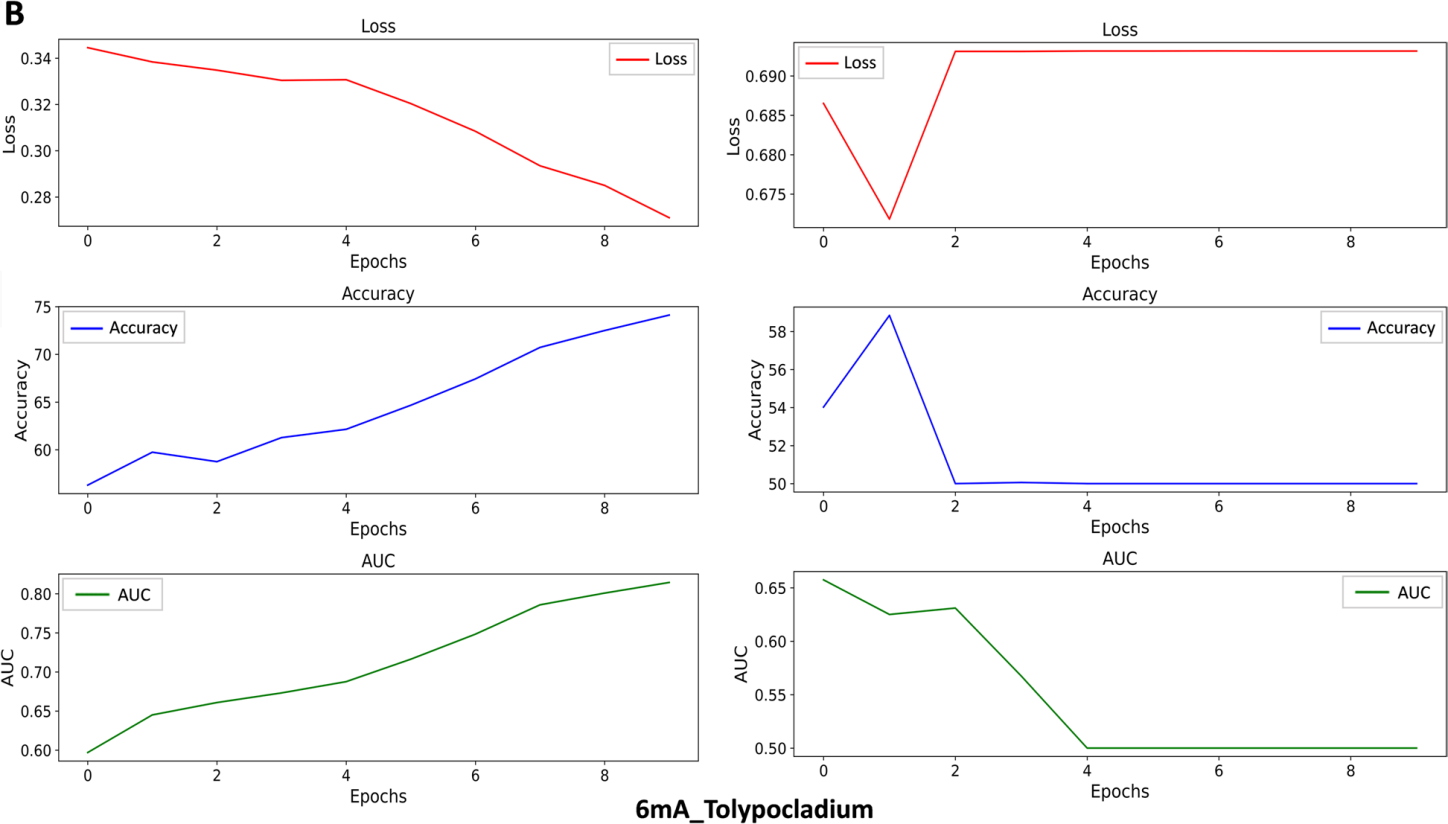
Performance comparison of the models on the 6mA_Tolypocladium dataset

To evaluate the stability and robustness of the StableDNAm model, we carried out comparative tests against the iDNA-ABF model, which has exhibited the second-best performance among existing solutions. After thorough testing across seventeen datasets, our StableDNAm model’s superior stability and robustness compared to the iDNA-ABF model, which exhibited inadequate stability, were evident. Figures 3 and 4 detail the experimental results for both models across all datasets, focusing on the training loss and accuracy (ACC) during the final 5 epochs. As depicted in Fig. 3, the loss heatmap for the iDNA-ABF model displays a significant increase in loss towards the latter training stages, highlighting its instability. Figure 4 offers a comparison of ACC between each dataset and the iDNA-ABF model. Its right subfigure uses a stacked bar chart to illustrate the cumulative ACC metric over the last five epochs, with higher stacking implying better metric stability during these epochs. This detailed analysis showcases the StableDNAm model’s potential for delivering robust and stable predictions across a variety of methylation datasets. We delve into the results from selected datasets, such as 4mC_Tolypocladium and 6mA_Tolypocladium, due to space constraints, while additional dataset results are available in the Supplementary files. In Figs. 5 and 6, the left and right subgraphs represent the performance of the StableDNAm and iDNA-ADF models, respectively. The iDNA-ABF model shows significant fluctuation in crucial metrics like ACC and AUC, and lacks a steady decline in the loss function. For instance, the results display the iDNA-ABF model’s AUC and ACC curves for the 4mC_Tolypocladium and 6mA_Tolypocladium datasets, which initially rise but eventually plateau around 0.5, without further improvement. This behavior can be attributed to the iDNA-ABF model’s overreliance on the pre-trained model, creating difficulties in fine-tuning it to specific datasets. Also, despite the fusion of $3-mer$ and $6-mer$ at varied scales during data input processing, the broad scale range may fail to capture intricate data characteristics, resulting in unstable predictions.

Contrarily, experimental results indicate that the StableDNAm model’s ACC and AUC metrics across all datasets consistently increase with more training iterations, accompanied by a decline in loss value. This behavior is a testament to the StableDNAm model’s exceptional stability and robustness across a range of datasets. Several factors contribute to this: Firstly, the StableDNAm model integrates $3-mer, 4-mer, 5-mer$, and $6-mer$ multi-scale, low-span features, enabling more comprehensive and robust feature extraction. Secondly, the 2D-SENET module adaptively adjusts sequence feature weights for different types of datasets. This adaptation allows the model to better understand and utilize critical input data features, enhancing model stability. Concurrently, the model can adapt to a variety of input scenarios, bolstering the robustness of prediction results. Lastly, the incorporation of a contrastive learning module reduces the impact of sparse data, enabling the model to more effectively distinguish between different class samples, thereby improving model stability and generalizability.

**Fig. 7:**
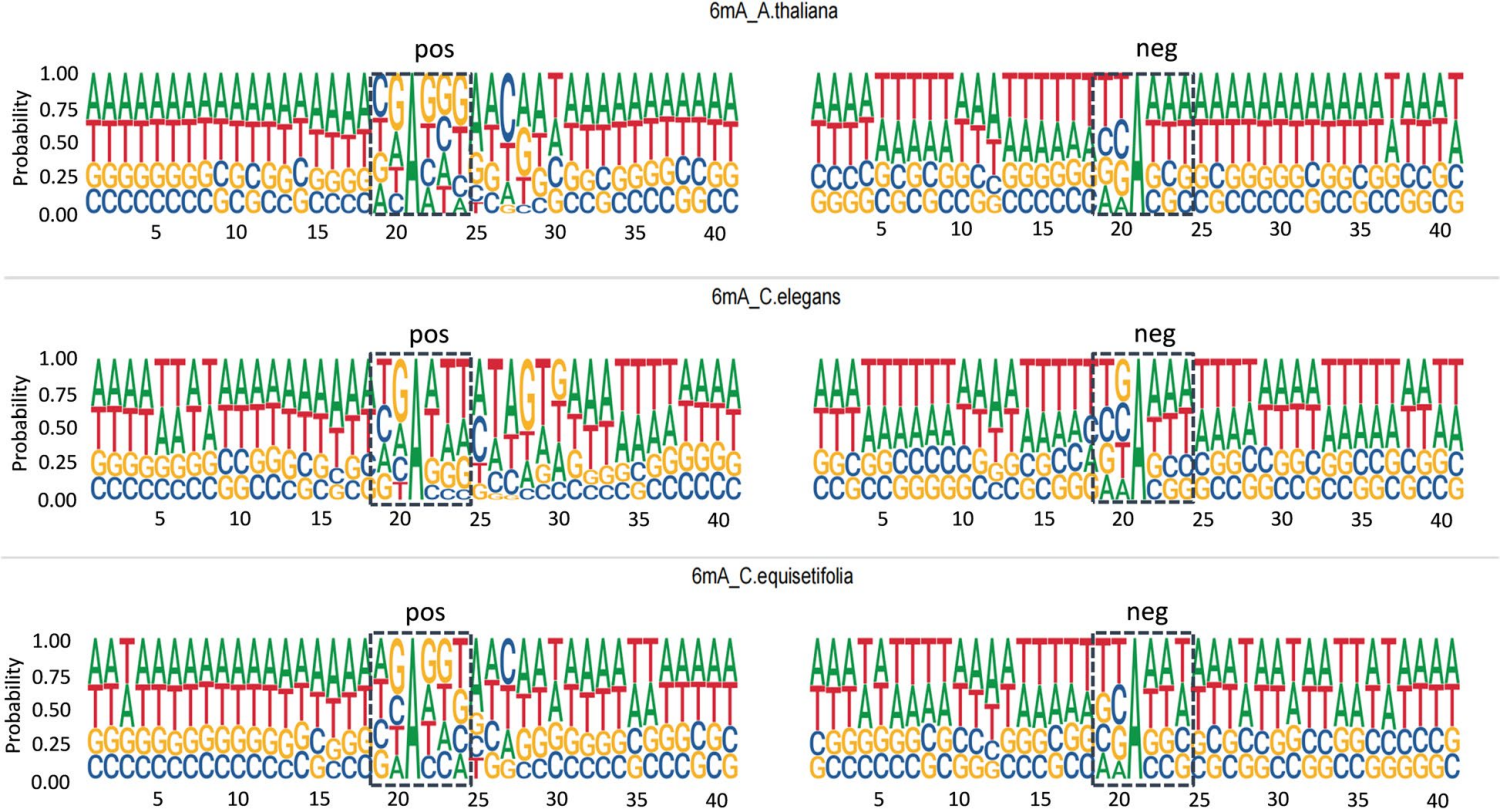
Conducting Motif Logos analysis on positive and negative datasets of three different species

In an effort to bolster our analysis, we segmented the DNA sequences into chunks of 41 base pairs, as illustrated in Fig. 7. This procedure facilitated the conduct of Motif logos analysis across both positive and negative datasets pertaining to three distinct species. Upon examination of the highlighted areas within the figure, one can observe marked differences. Our proposed model, which is fundamentally constructed on multiple $k-mer$s, leverages the latent information embedded within these sequences. Subsequently, a 2D-SENNT module is deployed to perform feature normalization, thereby further enhancing the accuracy and efficacy of the model.

### Ablation experiment

**Fig. 8:**
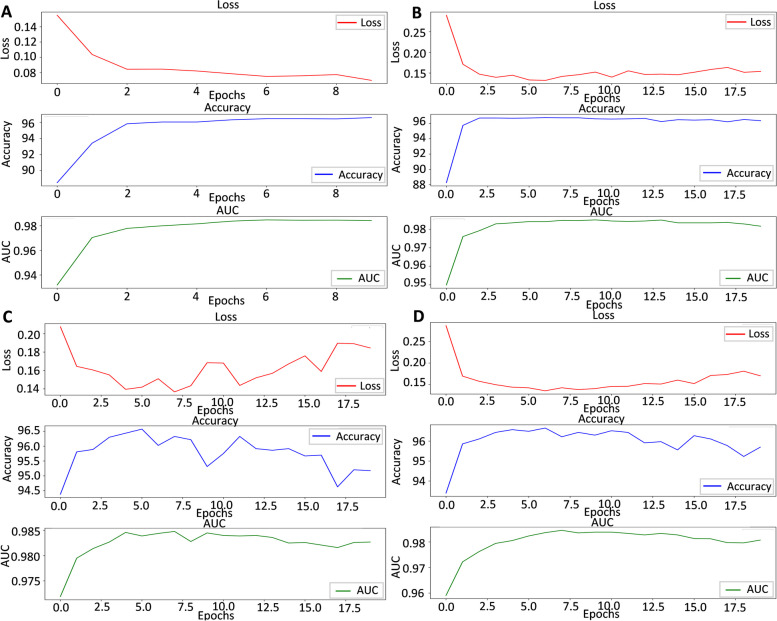
The results of ablation experiments, subfigure **A** shows the performance of the StableDNAm model, subfigure **B** shows the performance without the contrastive learning module, subfigure **C** shows the performance without the 2D-SNET module, and subfigure **D** shows the performance without the fusion module

To appraise the contributions of key modules within the StableDNAm model, we conducted several ablation experiments using the 5hmC_M.musculus dataset. Figure 8 presents the experimental outcomes, with subfigure A demonstrating the performance of the complete StableDNAm model, subfigure B displaying the performance with the contrastive learning module removed, subfigure C showing the performance without the 2D-SENET module, and subfigure D indicating the performance in the absence of the fusion module.

In subfigure A, the model’s loss value progressively decreases with the increase in training iterations on the 5hmC_M.musculus dataset, suggesting that the model’s adaptability to the test set improves with an increasing number of training rounds. Concomitantly, as the training advances, there is a steady increase in the *ACC* and *AUC* values, rising from 88.62% to 96.44%, and from 93.13% to 98.49% respectively. This upward trend reflects an enhancement in the model’s classification performance and its ability to classify samples more accurately.

Comparing subfigures A and B, we observe the model’s loss value continues to decrease, and the *ACC* and *AUC* values consistently increase, even without the contrastive learning module. However, in comparison to subfigure A, there is a slight fluctuation in the loss value during the latter phase of training in subfigure B, suggesting the model performs better wSubfigureontrastive learning module is included. This module helps the model more effectively discern differences and similarities between samples, thereby bolstering the model’s representation learning ability and generalization capacity.

The outcomes in subfigure C reveal that eliminating the 2D-SENNT module results in considerable fluctuations in the three curves, with no evidence of stabilization. Moreover, as the number of training rounds increase, the loss curve starts to ascend, while the *ACC* and *AUC* curves begin to descend, with the *ACC* curve exhibiting a more pronounced downward trend. These results suggest that the 2D-SENNT module significantly enhances the model’s convergence performance and elevates the *ACC* and *AUC* values. This module adaptively adjusts feature weights to suit different datasets, minimizes the interference of superfluous features, and enables fine-tuning of model parameters, thereby accommodating a wider range of downstream datasets.

As shown in subfigure D, starting from the 6-th iteration, the loss value begins to rise, corresponding to a gradual decrease in *ACC* and *AUC*. These curves also exhibit varying degrees of fluctuation. In contrast, when the fusion module is removed, the model performs poorly. In this study, we used a $3-mer$ scale for learning, and to comprehensively demonstrate the effect of integrating different scales, we conducted experiments, which can be found in the Supplementary file (k-mer.xlsx). From the results in this file, it can be observed that this module has the ability to fuse sequences of different scales, namely $3-mer, 4-mer, 5-mer$, and $6-mer$, which helps capture more complex features. At the same time, this low-span multi-scale design minimizes the loss of critical information.

A synthesis of the above findings reveals that performance without the contrastive learning module is superior to that without the fusion module, while performance without the fusion module exceeds that without the 2D-SENNT module. This indicates that the 2D-SENET module, which adaptively adjusts the features of different datasets, is the most pivotal component of the model, enhancing its stability and adaptability. Subsequently, the multi-scale and low-span feature fusion methodology, which captures complex features and reduces the loss of crucial dataset information, ranks as the second most significant component of the model. Finally, the contrastive learning module, which mitigates the impact of sparse data, is the third most important part of the model.

## Discussion

According to the analysis presented in Table 2, the StableDNAm and iDNA-ABF models, utilizing a pre-training strategy, demonstrate the top two performances across seventeen datasets. This exemplifies the significant effectiveness of the pre-training strategy in enhancing the efficiency of the models. Furthermore, the integration of a transformer encoder in our approach enables the models to autonomously extract DNA sequence characteristics, allowing it to adapt to the identification tasks of various types of DNA methylation datasets. The results from a unified dataset suggest that the proposed StableDNAm model can serve as a general tool for predicting methylation, while the iDNA-ABF model encounters challenges in identifying methylation sites. In-depth comparative analysis on seventeen individual datasets, as depicted in Figs. 5 and 6 and Supplementary files (All-curves.pdf), reveals that the iDNA-ABF model is excessively reliant on the pre-training model, which leads to difficulties in fine-tuning to accommodate the downstream dataset, resulting in highly variable performance. In contrast, our proposed StableDNAm model is capable of not only leveraging pre-training strategies for information accumulation but also adaptively fine-tuning in downstream datasets, thereby exhibiting efficient and stable performance. The observed robustness of the StableDNAm model could be attributed to the integration of a 2-D SENET module that adaptively refines sequence features, a multi-scale and low-span fusion module, and a contrastive learning strategy based on varying dropout rates. The influence of these three modules on the model performance was confirmed through ablation experiments. Nevertheless, the presented model comes with specific constraints. Its training demands over 16GB of GPU memory. Optimizing the model to operate effectively on a smaller GPU memory footprint will be focus of our upcoming research endeavors. Moreover, we aim to incorporate graph structure data of DNA and additional information to enhance the accuracy of DNA methylation prediction.

## Conclusions

This study delves into various models for DNA methylation prediction, revealing that the performance of existing models is often hampered by limited stability and scalability. As a result, we introduce the StableDNAm model, a new model capable of predicting various types of DNA methylation using a Transformer encoder and a contrastive learning strategy. Our model incorporates a unique low-span, multi-scale feature fusion strategy to integrate $3-mer, 4-mer, 5-mer$, and $6-mer$ sequence features, thereby capturing more complex features. Simultaneously, this low-span, multi-scale design aids in minimizing the loss of vital information. Additionally, we crafted a novel 2D-SENNT module that adaptively adjusts sequence feature weights in different datasets, further bolstering the model’s stability and scalability. The contrastive learning module embedded within our model also addresses the issue of sparse data, mitigating its impact. We conducted exhaustive verification experiments across seventeen diverse methylation datasets, alongside a comparison with a unified dataset composed of these seventeen datasets. Our research findings affirm that the StableDNAm model is a general, stable, and effective instrument for DNA methylation prediction. It holds substantial promise for providing invaluable assistance in future methylation-related research and analyses.

## Methods

### Model architecture

**Fig. 9:**
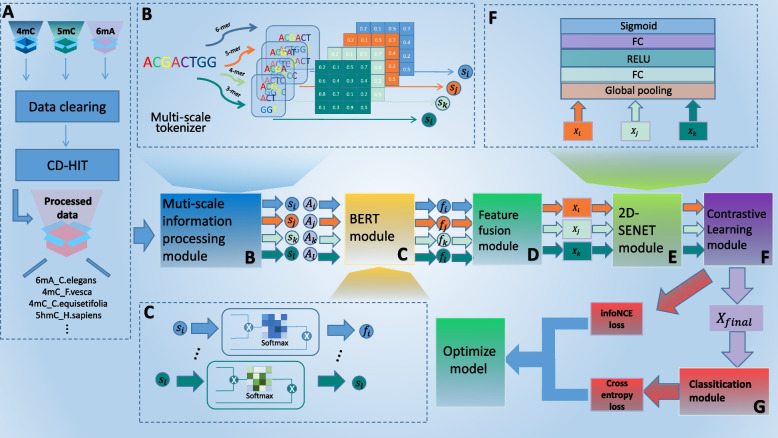
The overall architecture and workflow of the StableDNAm model. **A** represents the data collection process described in Datasets section. The model architecture of the StableDNAm model mainly consists of six modules: **B** multi-scale data processing module, **C** BERT encoder module, **D** full feature fusion module, **E** 2D-SENET module, **F** contrastive learning module, and **G** classification module

This paper presents StableDNAm, a novel deep-learning model built on the Transformer architecture, specifically designed for the reliable and efficient identification of DNA methylation. Our model is characterized by three strategic components: feature fusion, feature correction, and contrastive learning. Figure 9 provides a detailed visual representation of the proposed StableDNAm model’s architecture and workflow. (A) The initial module revolves around data collection. (B) In this module, the representation of the sequence is computed across four scales ($3-mer, 4-mer, 5-mer, 6-mer$). To accommodate the diverse range of input DNA sequences stemming from various datasets, we implement a four-scale approach to derive a more complex feature set and thus enhance adaptability across different datasets. (C) Following this, four BERT encoders are deployed to extract features at these four scales. (D) This module integrates the multi-scale embeddings provided by the previous module. (E) In this module, the output from the 2D-SENET module undergoes a feature weight adjustment. This key step adaptively corrects the fused features respective to different datasets, contributing significantly to the model’s stability and scalability. (F) The StableDNAm model employs a contrastive learning strategy to formulate positive and negative samples, enhancing the differentiation and similarity within the original data. This effectively mitigates the adverse effects of sparse datasets on the model. (G) Finally, the model stacks multiple fully connected layers to predict the methylation status of the input DNA sequence. Each of these modules is described in further detail in the ensuing sections.

### Multi-scale and low-span features

To enhance the applicability of the model to various DNA methylation datasets, we construct the initial DNA sequence embedding utilizing multi-scale and low-span features ($3-mer, 4-mer, 5-mer, 6-mer$). This strategy allows for a more effective dataset adaptation and a more comprehensive sample information capture, while minimizing the loss of lesser important details. Consequently, it ensures the model’s performance remains both stable and scalable. In previous research (ABF), only two scales ($3-mer$ and $6-mer$) were utilized as initial DNA sequence features. This method, due to its large span, could only account for information at longer ($6-mer$) and shorter ($3-mer$) scales.

The $k-mer$ process mainly revolves around constructing the DNA sequence’s initial features. Specifically, the DNA sequence is segmented into several subsequences, each of length *k* and a step size of 1; these subsequences are referred to as “tokens”. In a manner akin to Natural Language Processing (NLP), this method initially segments the sequence and treats each subsequence as a “word”. For instance, given a DNA sequence “GGTCCA”, its corresponding $3-mer$ token list would be [“GGT”, “GTC”, “TCC”, “CCA”]. Coupled with a few specific tokens, these establish the comprehensive initial features of the DNA sequence based on the $k-mer$.

### BERT encoder

BERT is a cornerstone module predominantly used in natural language processing (NLP). Its main role is to utilize the self-attention mechanism inherent in the Transformer architecture for text processing. BERT proves effective in various language tasks as it can comprehend text in context with high accuracy. In this research, we treat DNA sequences as text sequences, enabling their efficient processing with the BERT encoder. Pre-training on large-scale datasets typically results in superior performance on subsequent tasks. Correspondingly, the StableDNAm model also employs a pre-training model called DNABERT [42], rendering it more apt for handling downstream datasets.

A DNA sequence is composed of numerous bases with a certain degree of correlation, which allows gene fragments to execute specific functions. Studying gene correlations based on a single base provides limited insight. BERT proves highly compatible with such DNA sequences, treated as text, as it can expose connections between multiple adjacent bases. BERT employs a stacked Transformer architecture, primarily encompassing multi-head attention and feedforward network modules. For the initial feature of the DNA sequence of $k-mer$, the attention mechanism computation proceeds as follows:

7 $$\begin{aligned} Q=XW_1, K=XW_2, V=XW_3, \end{aligned}$$

8 $$\begin{aligned} f_{Att}(Q,K,V)=softmax(QK^T/\sqrt{d_k})V, \end{aligned}$$

where $X \in \times d_1$ represents the initial characteristics of the DNA sequence, *n* represents the number of initial tokens of the DNA sequence, and $d_1$ represents the dimension of the token. $Q, K, V\in n \times d_2$represent *Query*, *Key*, and *Value* matrices respectively, $W_1$, $W_2$, and $W_3$ represent the corresponding weight matrix, and $d_2$ represents the dimension. $f_{Att}$ represents the attention function. The multi-head attention mechanism can be expressed as:

9 $$\begin{aligned} h_i=f_{Att}(XW_{1,i},XW_{2,i},XW_{3,i}), i=1,2,...,H, \end{aligned}$$

10 $$\begin{aligned} f_{multiAtt}(Q,K,V)=[h_1,h_2,...,h_H]W_m, \end{aligned}$$

where $XW_{1,i}, XW_{2,i}$ and $XW_{3,i}$ respectively represent the linear transformation matrix of *Query*, *Key*, and *Value* of the $j-th$ attention head, and *H* represents the total number of attention heads. Then, the results of the multi-head attention are mapped through the linear transformation matrix $W_m$.

#### Feature fusion

The DNA sequence undergoes processing through four BERT encoders, each extracting distinctive features based on four scales ($3-mer, 4-mer, 5-mer, 6-mer$). Smaller scales capture local specific information, while larger scales represent longer dependencies between bases. Fusing features based on these different scales integrates both long-dependent and short-dependent information. Given that different levels of information may hold varying significance for different datasets, it is crucial to reasonably fuse multi-layered information. For the output results of the four BERT encoders, we utilize a linear layer for weighted fusion instead of traditional concatenation. The detailed computation is as follows:

11 $$\begin{aligned} F=\sigma (\omega _1\cdot h_{3mer}+\omega _2\cdot h_{4mer}+\omega _3\cdot h_{5mer}+\omega _4\cdot h_{6mer}), \end{aligned}$$

12 $$\begin{aligned} h_{fusion}=F\cdot (h_{3mer}+h_{4mer}+h_{5mer}+h_{6mer}), \end{aligned}$$

where $h_{fusion}$ represents the fused features, *F* represents the calculated mapping matrix, and $h_{3mer}$, $h_{4mer}$, $h_{5mer}$, and $h_{6mer}$ represent the features extracted by DNA sequences using BERT encoders based on $3-mer$, $4-mer$, $5-mer$, and $6-mer$, respectively. This module considers the importance of different scales, but does not consider the importance of features, which may lead to unstable performance of the model when processing different datasets. Therefore, this study subsequently designed a feature correction module to adaptively adjust the weight of features.

### Feature adaptive adjustment (2D-SENET)

**Fig. 10:**
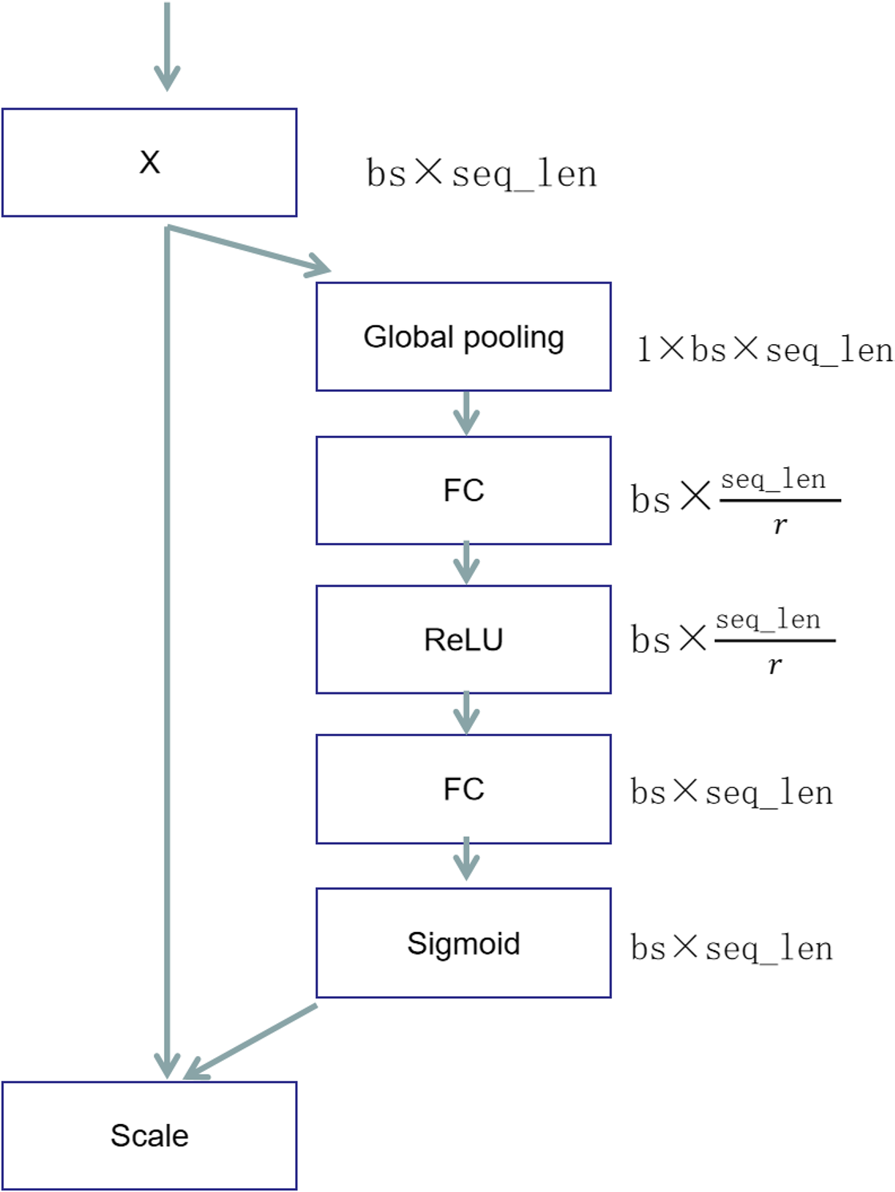
Figure of 2-D SENET

SENET, a highly efficient model in the field of image processing, is capable of adaptively adjusting feature weights based on feature channels [43]. It effectively extracts more important features while downplaying the less significant ones. Due to its excellent performance on numerous image tasks and its relatively lightweight nature, SENET is widely used in the field of imaging. Traditionally, SENET targets two-dimensional or three-dimensional images, and its application in sequence processing remains limited. In this study, we made suitable modifications to make SENET compatible with sequence feature correction. Specifically, as shown in Fig. 10, we treat the length of the sequence as a channel. By utilizing average pooling and multiple fully connected layers, where average pooling helps extract local features by taking the average of features within each pooling window, emphasizing local information and highlighting useful details, this mechanism is particularly suitable for adjusting the importance of sequence features. It can adaptively adjust the weights of the features obtained through feature fusion in Feature fusion section. As a result, our model can accommodate different types of methylation datasets, enhancing model stability and scalability.

### Contrastive learning

Contrastive learning strategies are widely employed in training deep learning models. They are particularly adept at discerning the similarities and differences between samples, and can help alleviate the effects of sparse samples on the performance of the model. In our study, each DNA sequence is inputted into 12 BERT encoders. The initial features of each DNA sequence at various scales ($3-mer, 4-mer, 5-mer, 6-mer$) are simultaneously fed into BERT encoders functioning at three distinct dropout rates (0.15, 0.3, and 0.9). Following this, the feature fusion module integrates the features at the four scales ($3-mer, 4-mer, 5-mer, 6-mer$) at the same dropout rate, yielding three groups of fused features with different dropout rates. These are then adaptively fine-tuned through the 2D-SENET module.

As indicated in previous research [44], representations with similar dropout rates manifest more similarities, while those with higher dropout rates demonstrate more significant differences. Operating under this premise, we utilize the InfoNCE function [45] to compute the loss of contrastive learning. The corresponding formula is as follows:

13 $$\begin{aligned} L_{infoNCE}=-EX\left[ log \frac{e^{sim(z_{r,i},z_{s,i})/\tau }}{\sum \nolimits _{j=1}^{N}e^{sim(z_{r,i},z_{t,j})/\tau }} \right] , \end{aligned}$$

where $z_ {r, i}$, $z_ {s, i}$, and $z_ {t, i}$ represent the embeddings obtained from sample $x_i$ through BERT encoders with dropout rates of 0.15, 0.30, and 0.90, respectively, and *sim*(., .) denotes the similarity function, which in this case is cosine similarity. $\tau$ stands for the temperature parameter. To maximize the mutual information between positive sample pairs and minimize it between negative sample pairs, relevant information is often integrated when constructing different positive and negative samples. Changes in the dropout rates lead to the learning of different features in sparse datasets. By constructing these samples three times and incorporating them into the InfoNCE loss function, we maximize the mutual information among similar samples. This can be understood as emphasizing important features, and this emphasis step mitigates the impact of sparse datasets on the model.

### Classification

This module mainly comprises three components: a fully connected layer, an activation function, and binary cross-entropy (BCE) loss. Once we acquire the final feature $X_{\text {final}}$ of the DNA sequence, we undertake feature mapping via the fully connected layer. For this model, we select the RELU activation function. In the final fully connected layer, its dimension is set to 2. After a sequence of transformations are carried out on $X_{\text {final}}$ by the classification module, a two-dimensional vector is eventually generated, representing the probability of the two classes. Using the two-dimensional vector of the positive and negative samples, we employ the BCE loss function to calculate the loss of these samples. The formula for BCE loss is as follows:

14 $$\begin{aligned} L_{BCE}(p,y|x,\theta )=ylog p-(1-y)log (1-p), \end{aligned}$$

where *x* represents the current DNA sequence, *p* represents the predicted score, *y* represents the true label, and $\theta$ represents all parameters of the model.

### Supplementary Information

**Additional file 1.**

## Data Availability

Our code and data are publicly available at: https://github.com/wrab12/StableDNAm. It’s easy to learn how to use our model through the readme file on GitHub. Additionally, step-by-step instructions on how to use the proposed model can be found in the Supplementary file (example.md).
