# Supplementary material for: StableDNAm: towards a stable and efficient model for predicting DNA methylation based on adaptive feature correction learning
Source: BMC Genomics. 2023 Dec 5;24:742. doi: 10.1186/s12864-023-09802-7 (PMC10698904; doi:10.1186/s12864-023-09802-7)

4mC_C.equisetifolia

StableDNAm

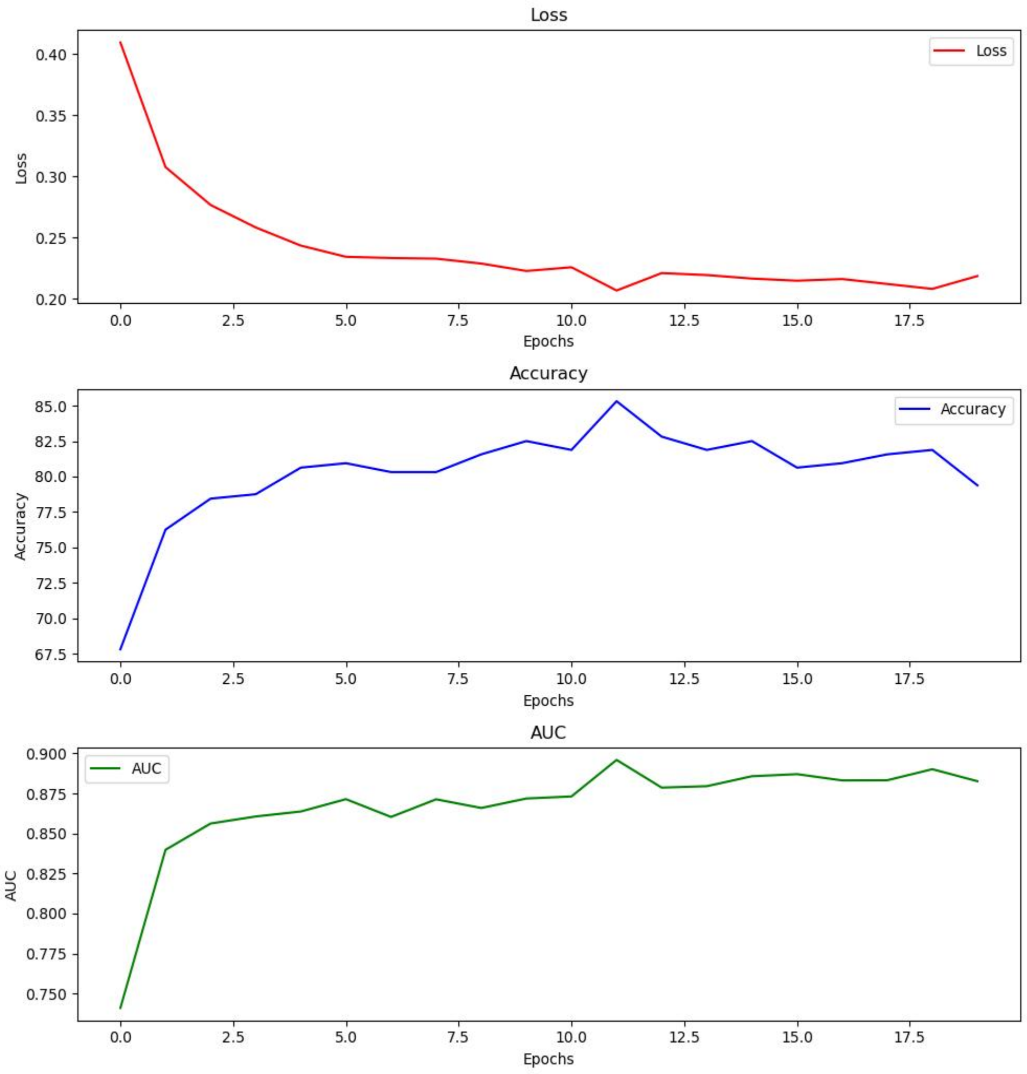

iDNA-ABF

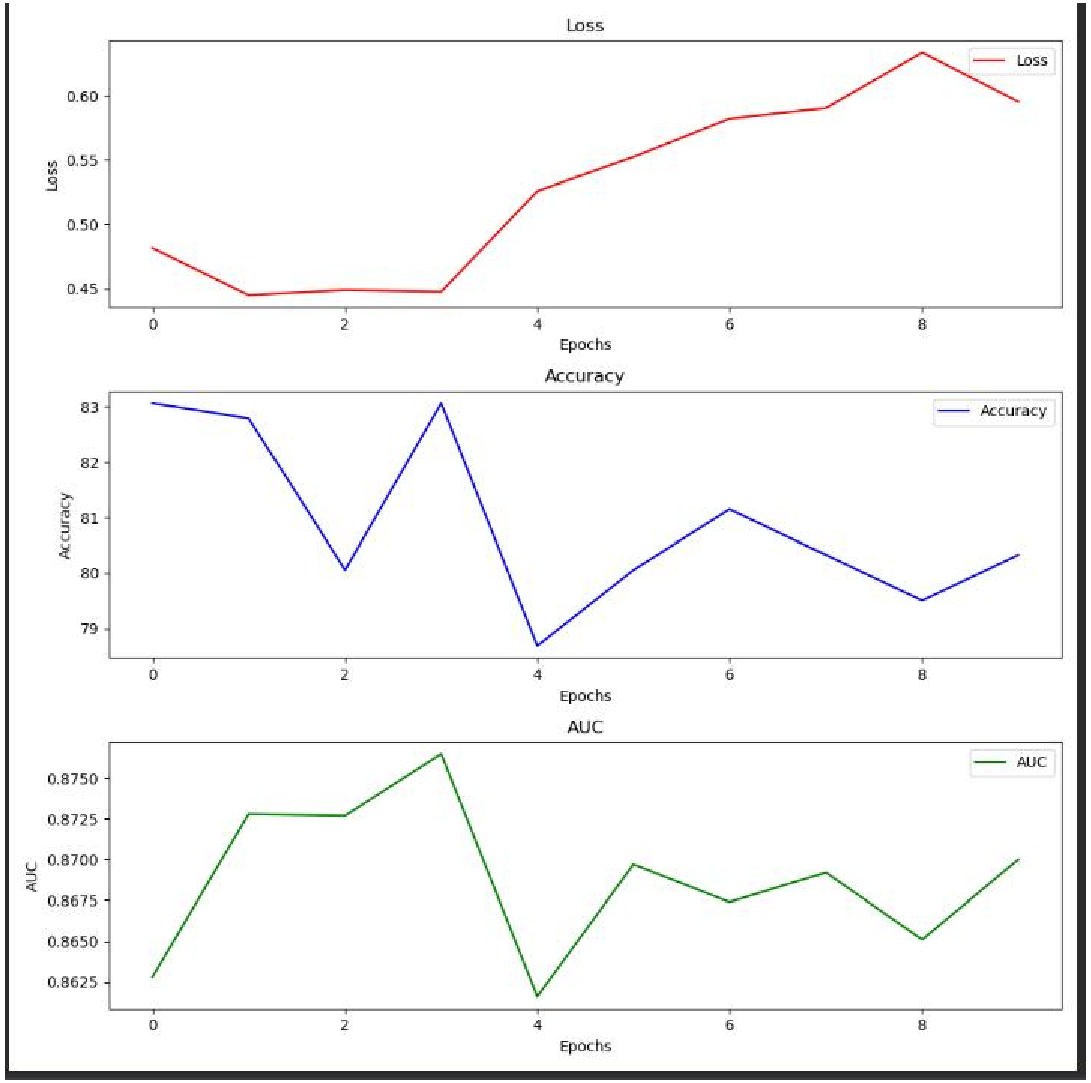

4mC_F.vesca

StableDNAm

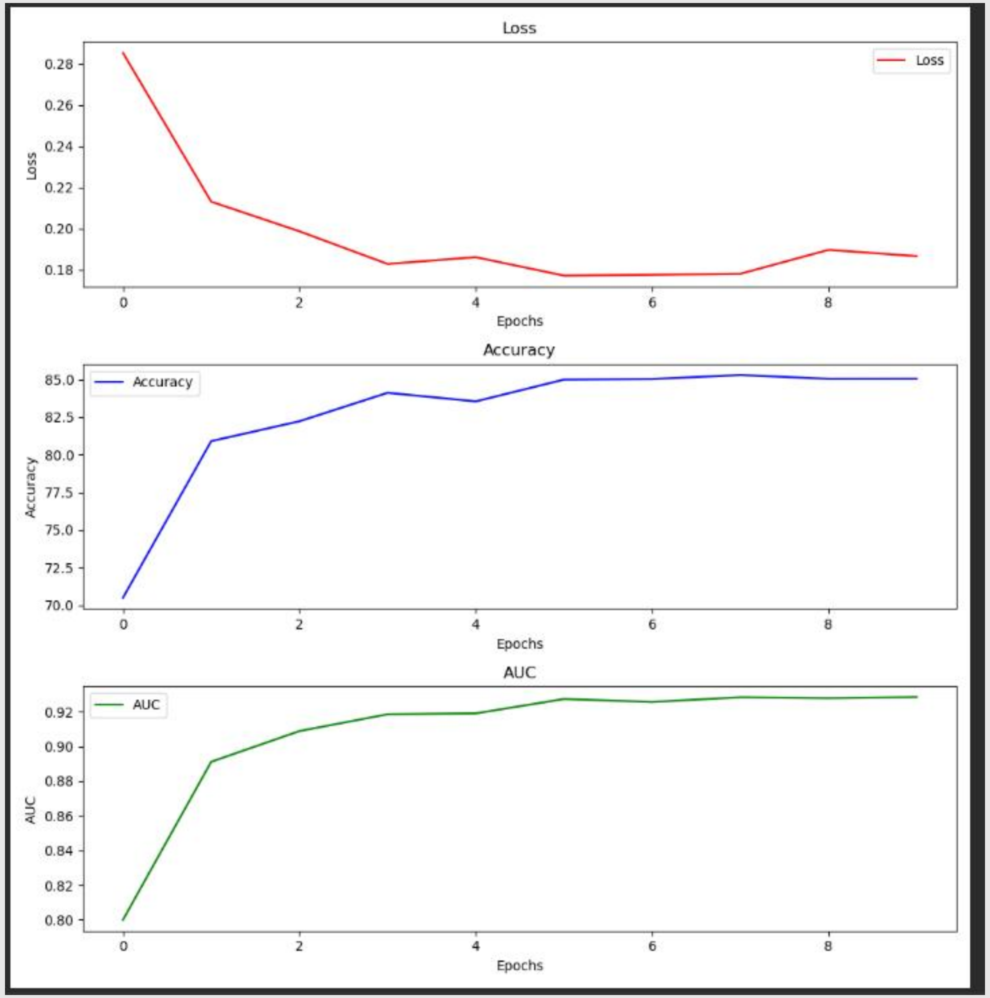

iDNA-ABF

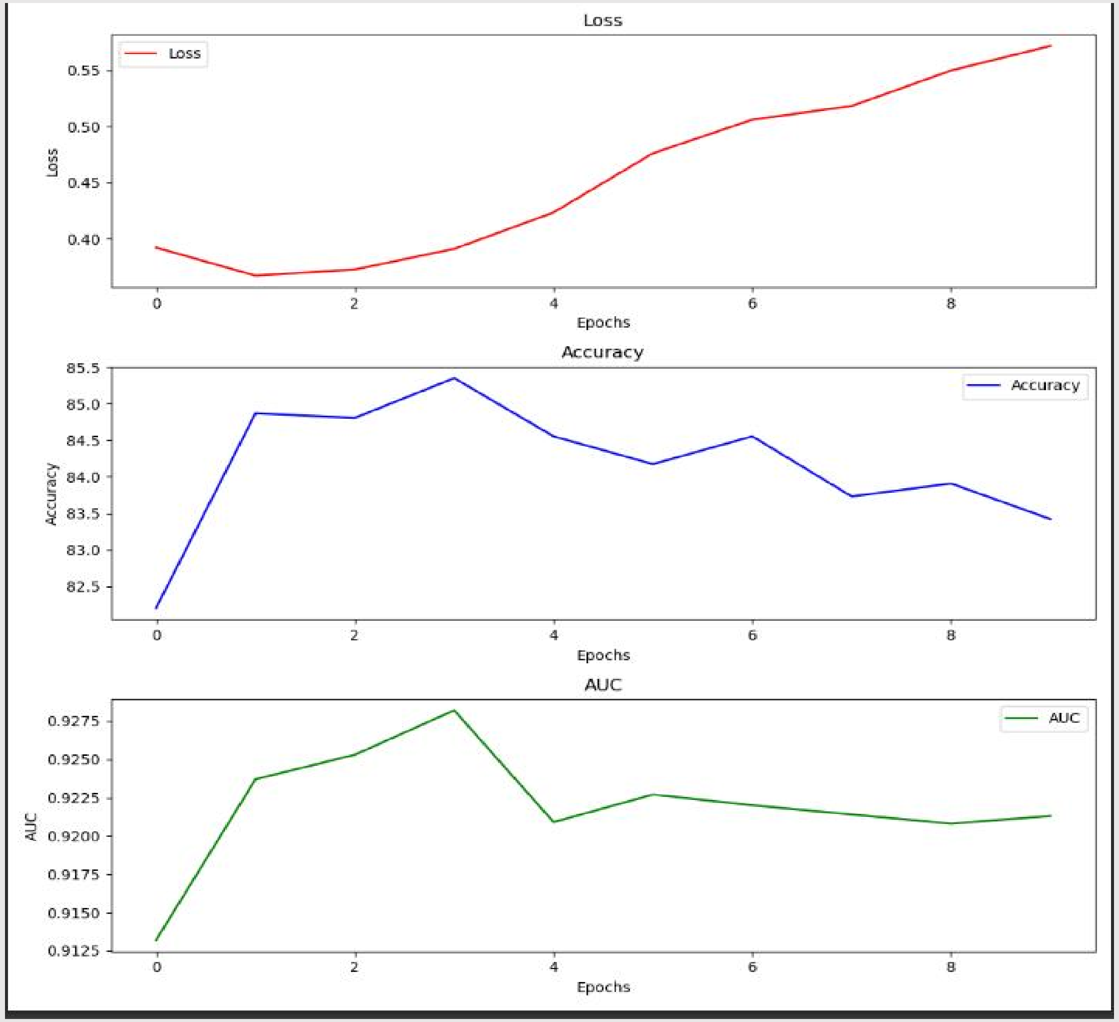

4mC_S.cerevisiae

StableDNAm

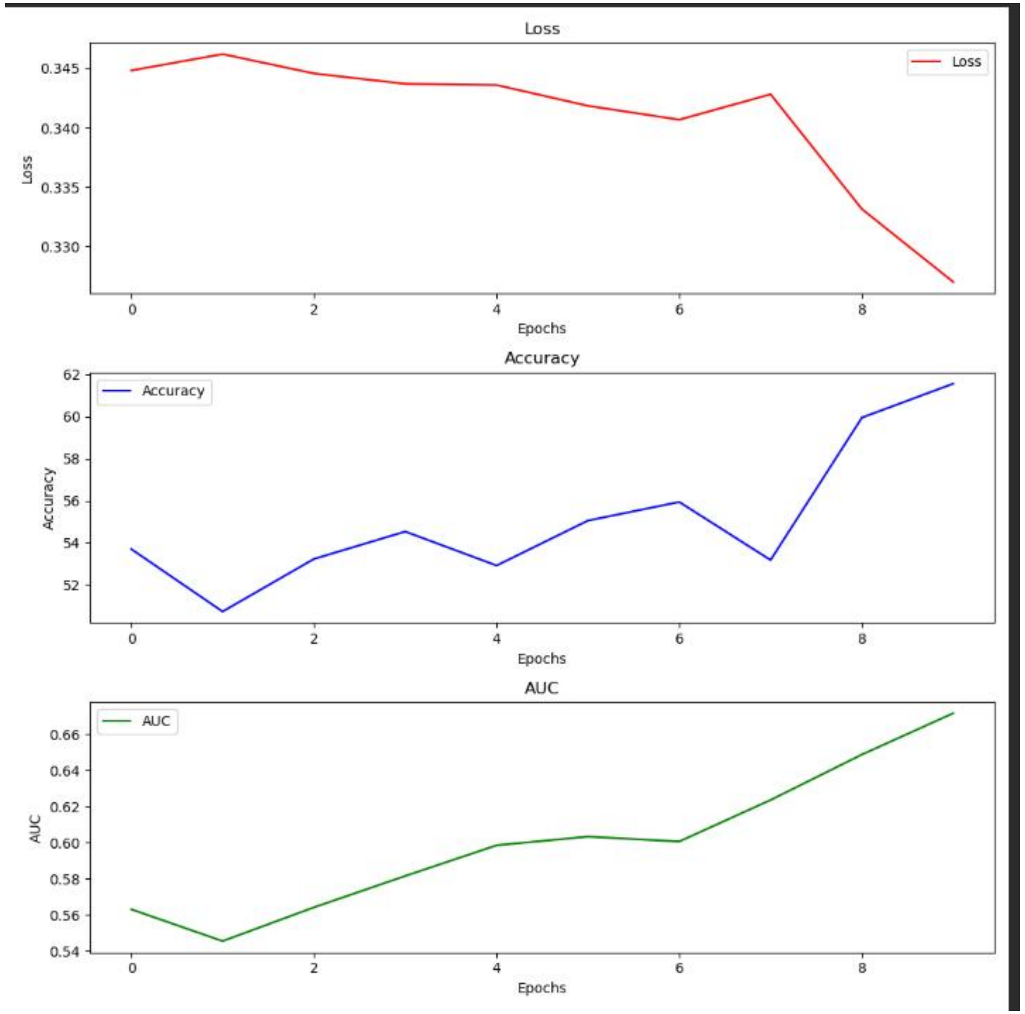

iDNA-ABF

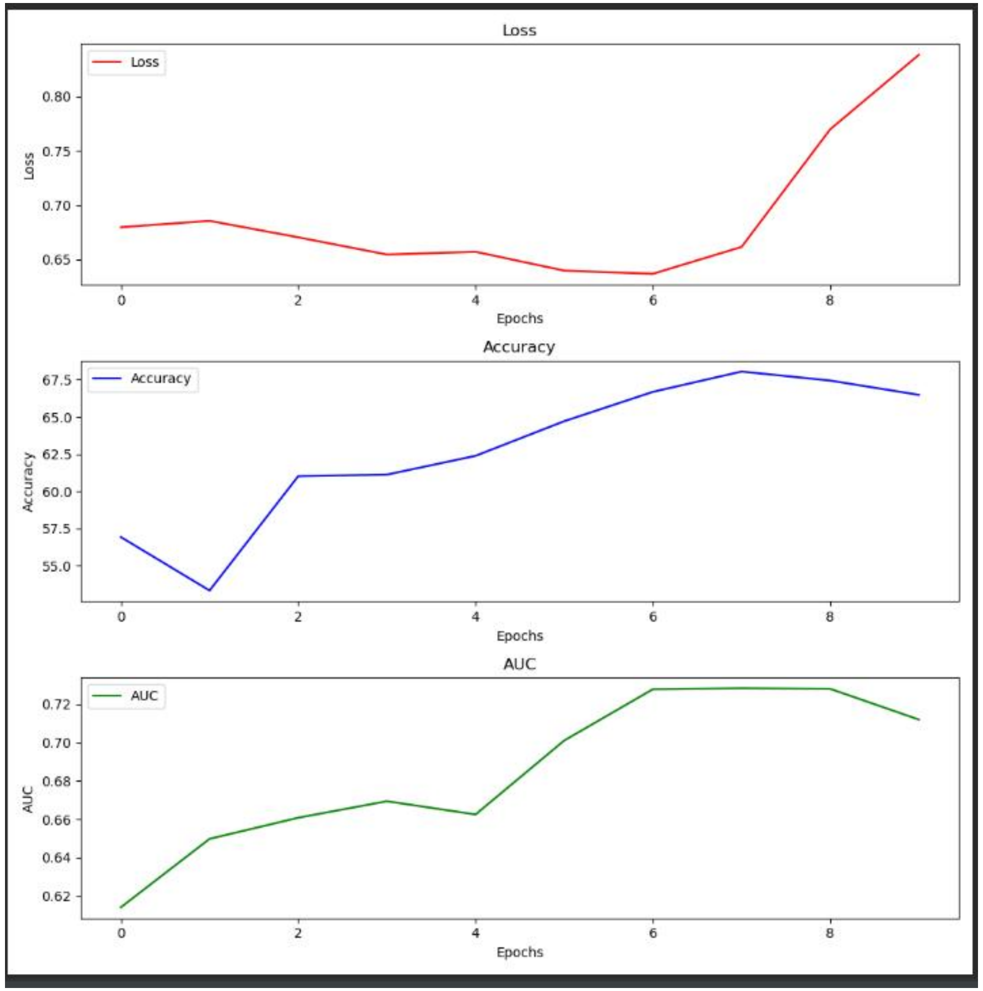

4mC_Tolypocladium

StableDNAm

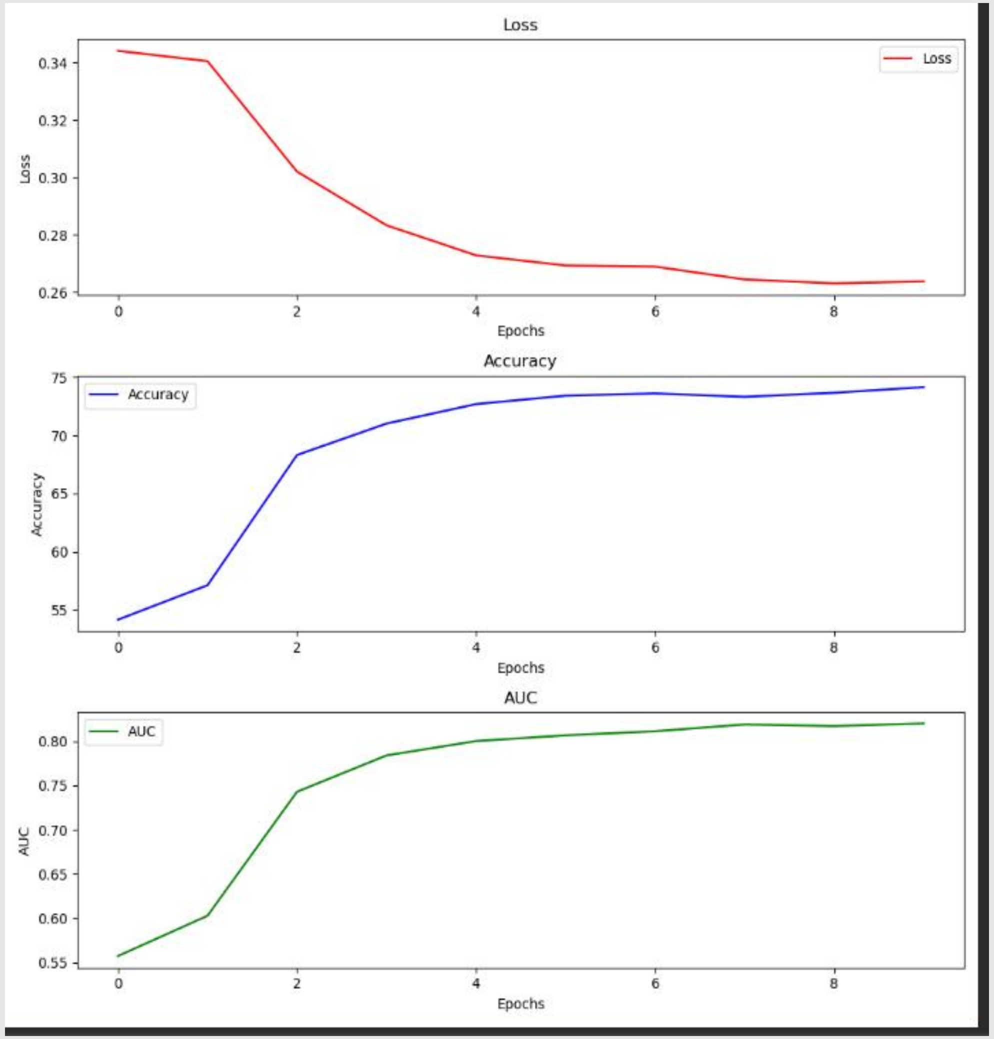

iDNA-ABF

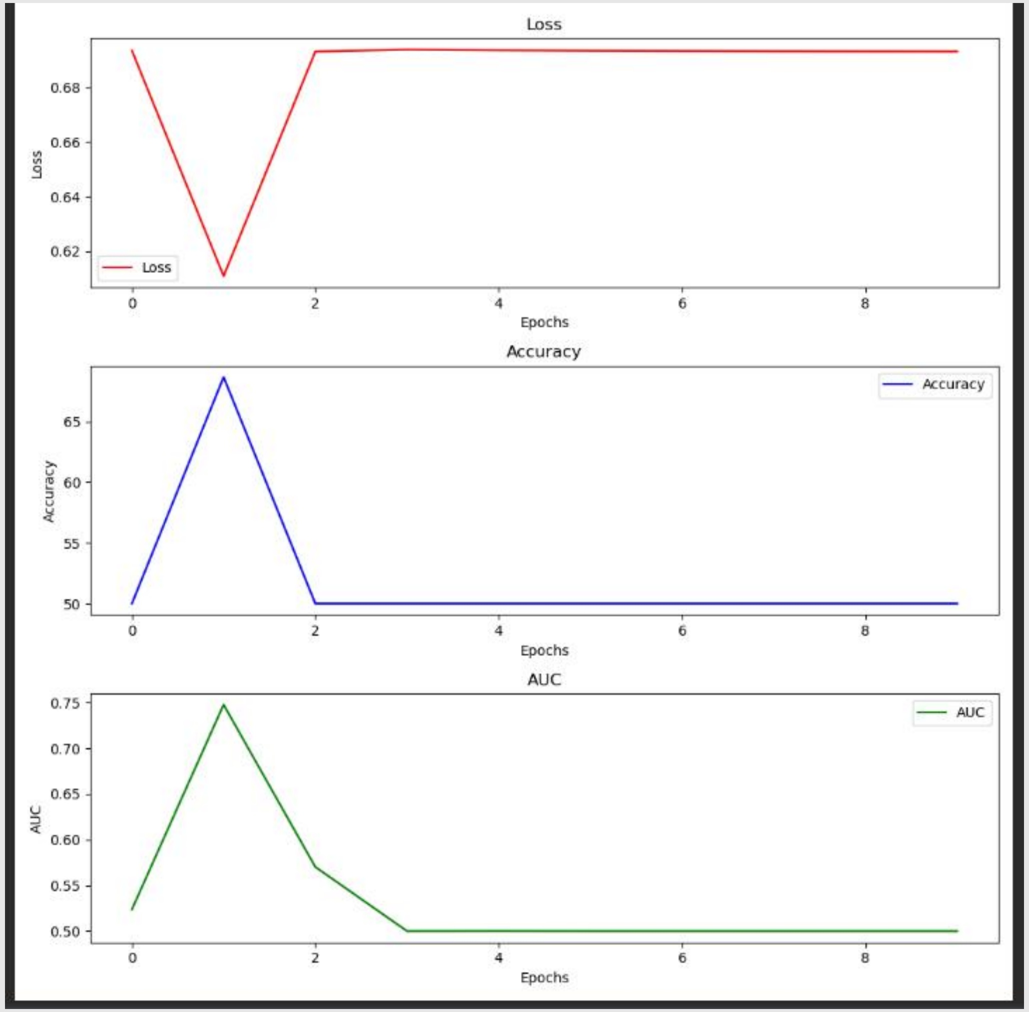

5hmC_H.sapiens

StableDNAm

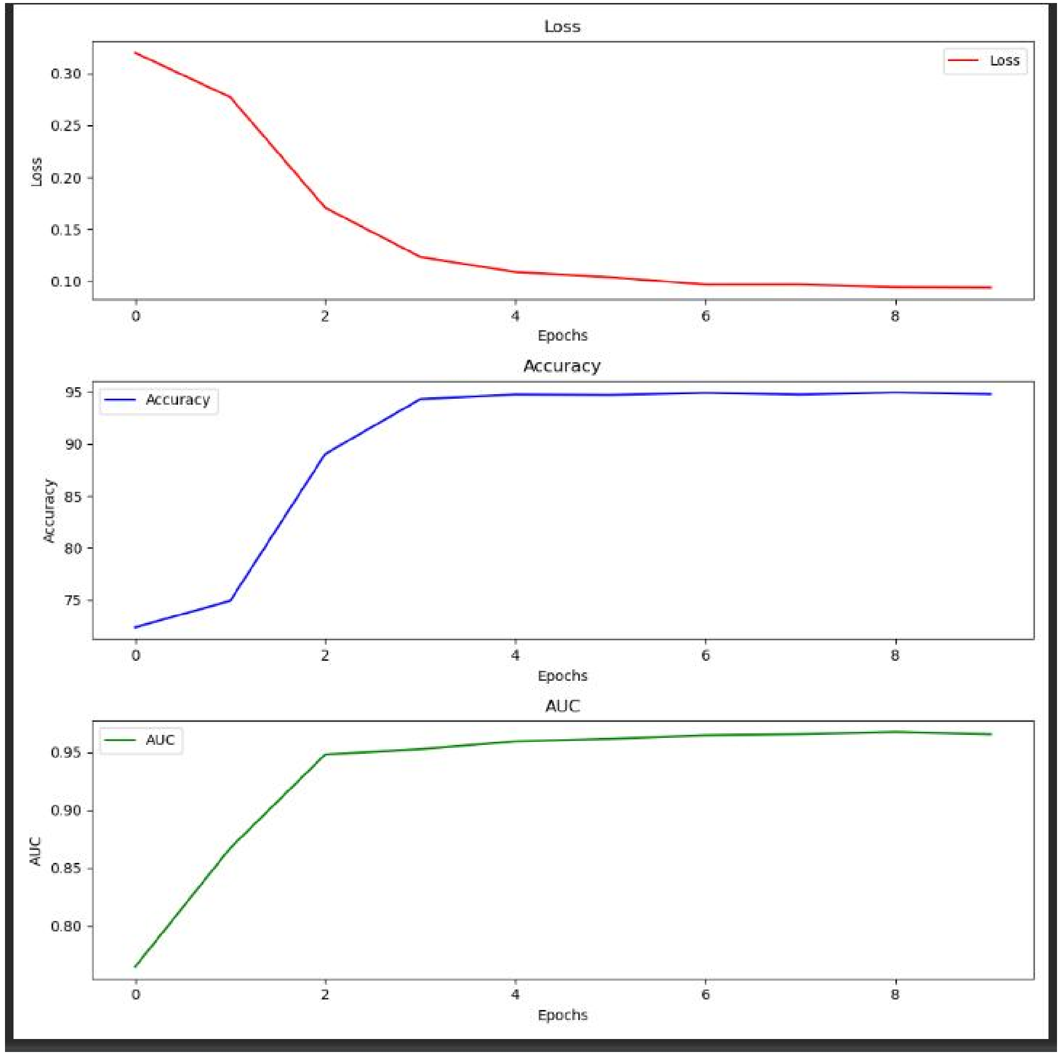

iDNA-ABF

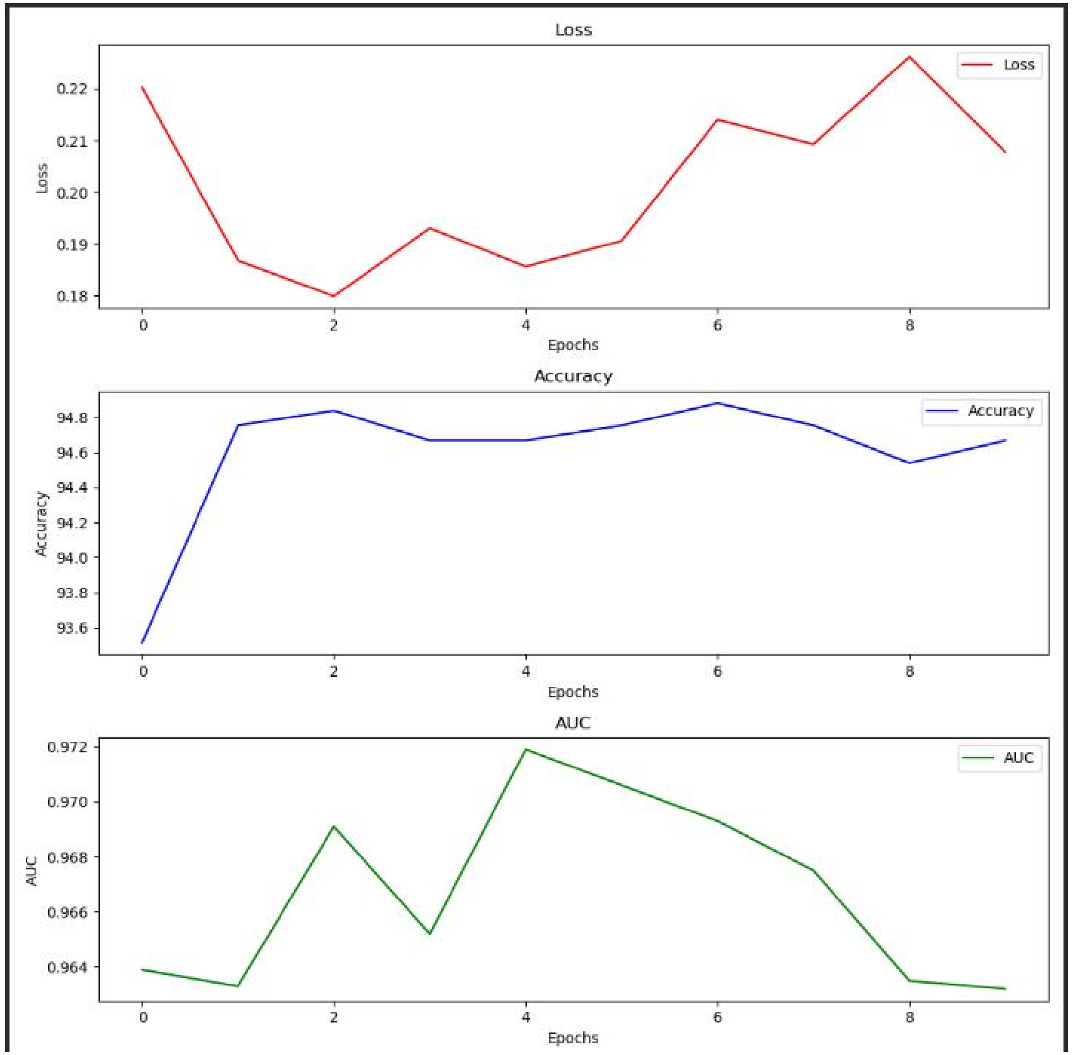

5hmC_M.musculus

StableDNAm

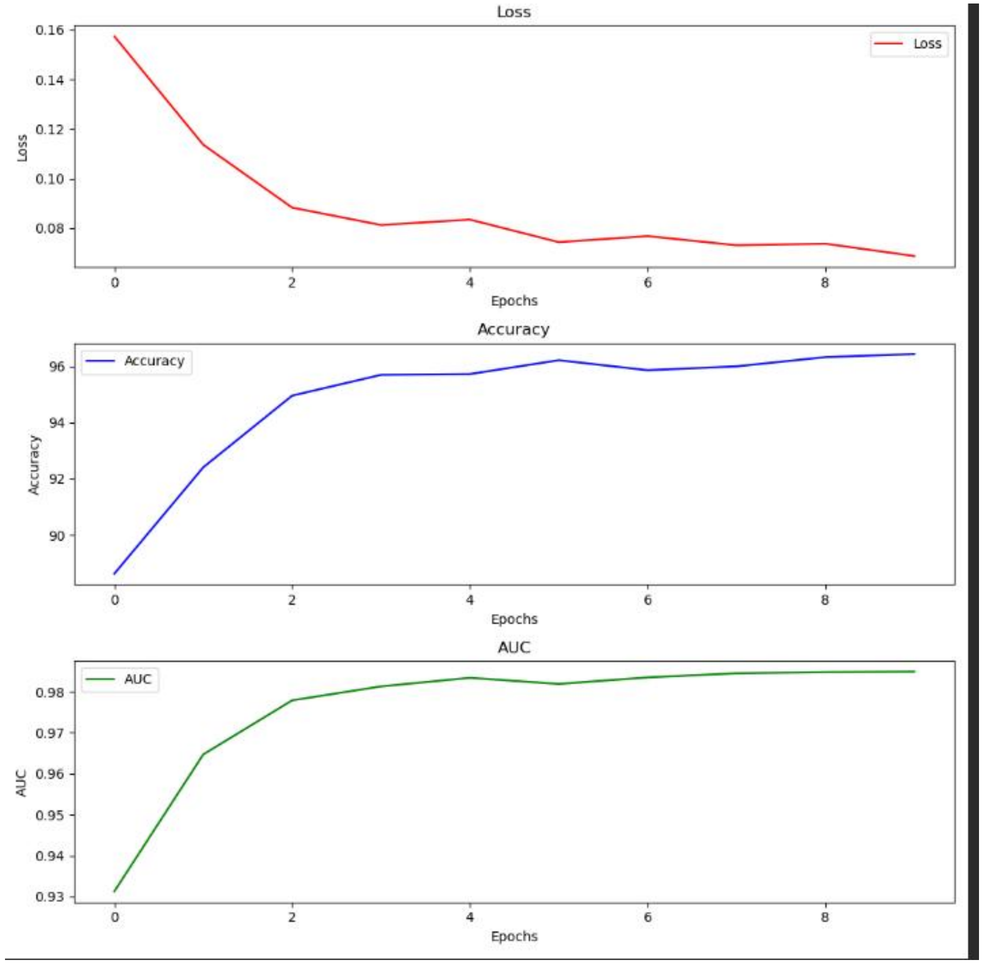

iDNA-ABF

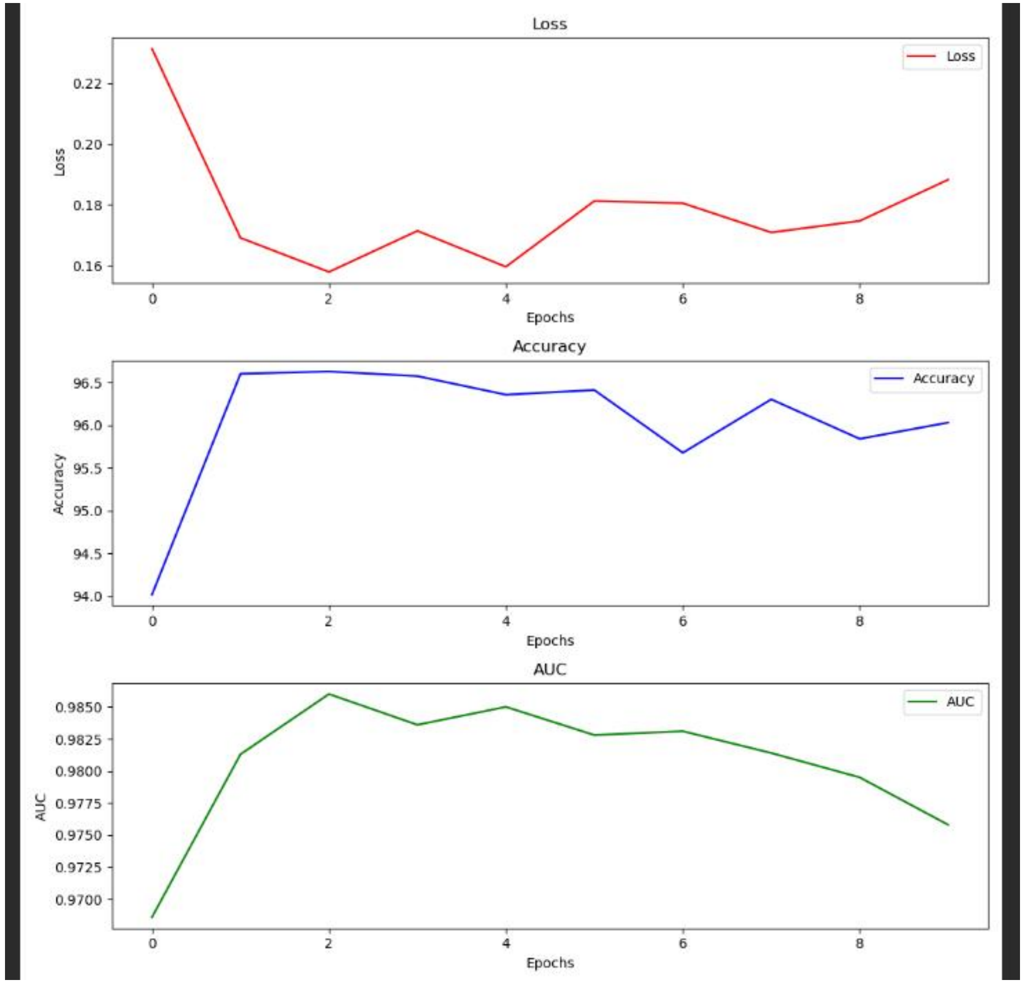

6mA_A.thaliana

StableDNAm

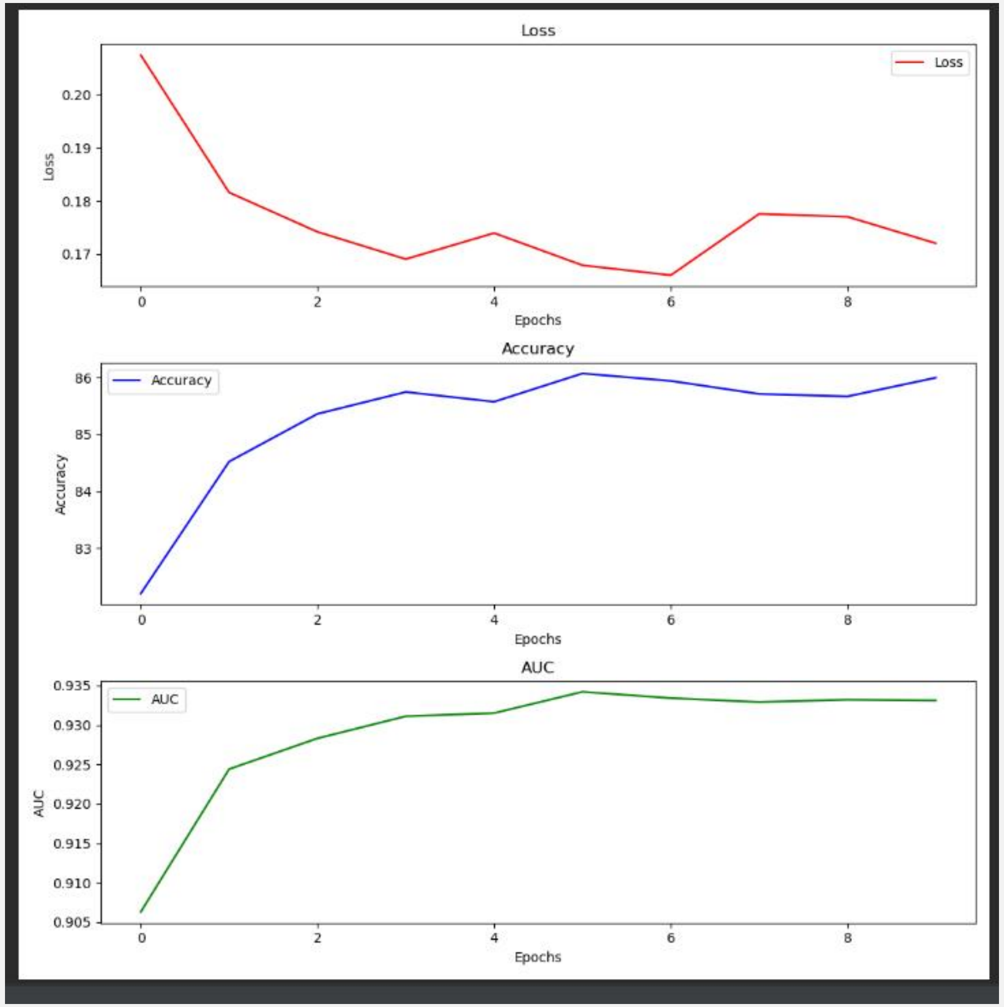

iDNA-ABF

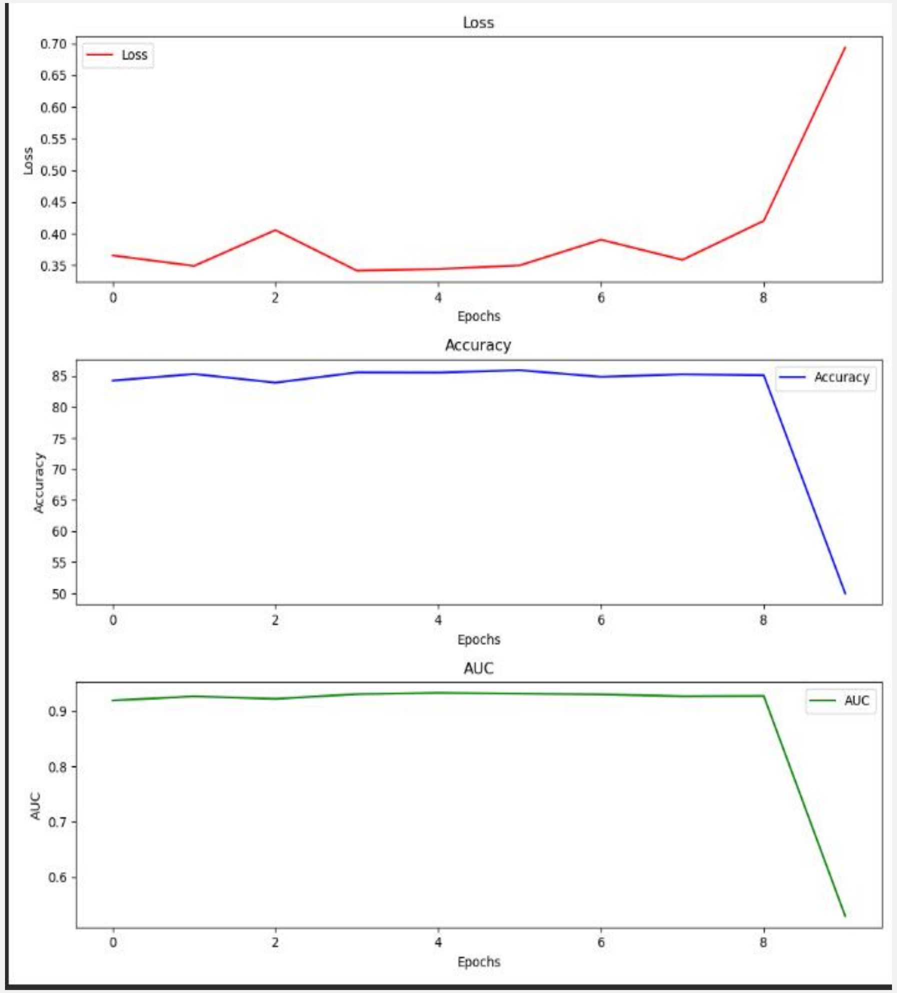

6mA_C.elegans

StableDNAm

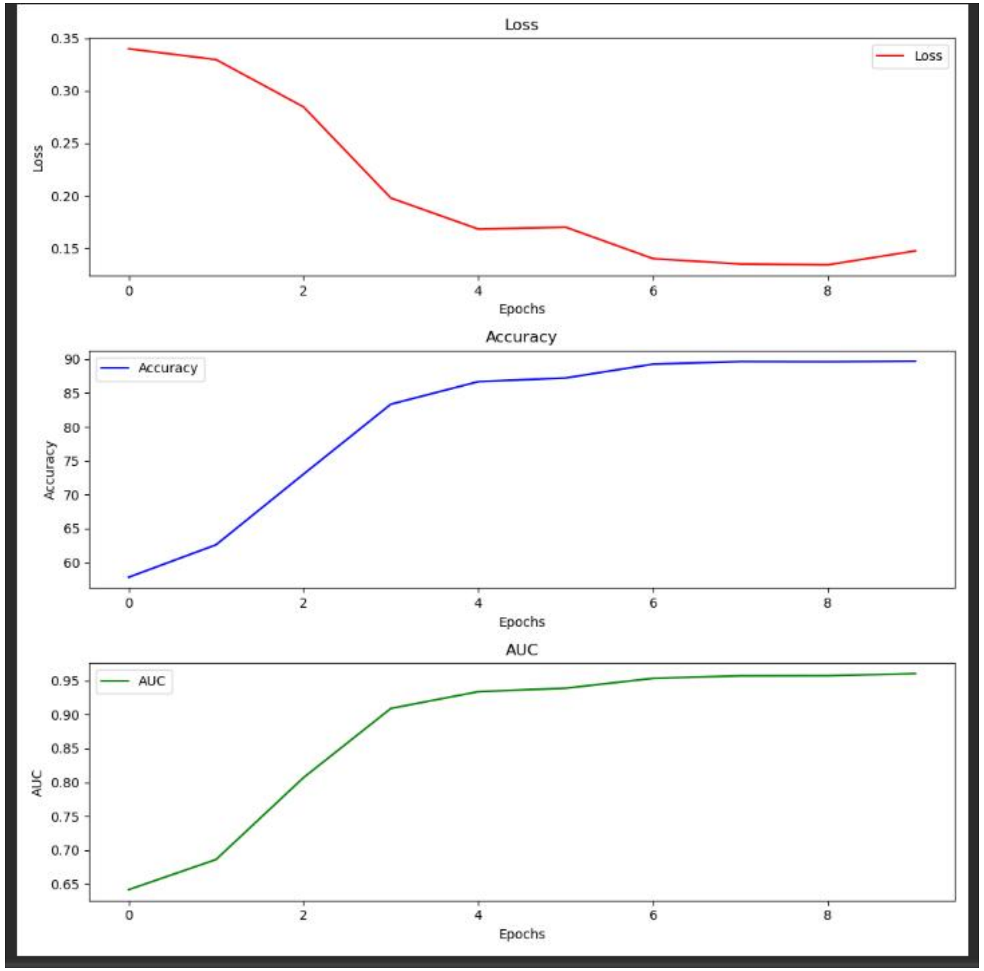

iDNA-ABF

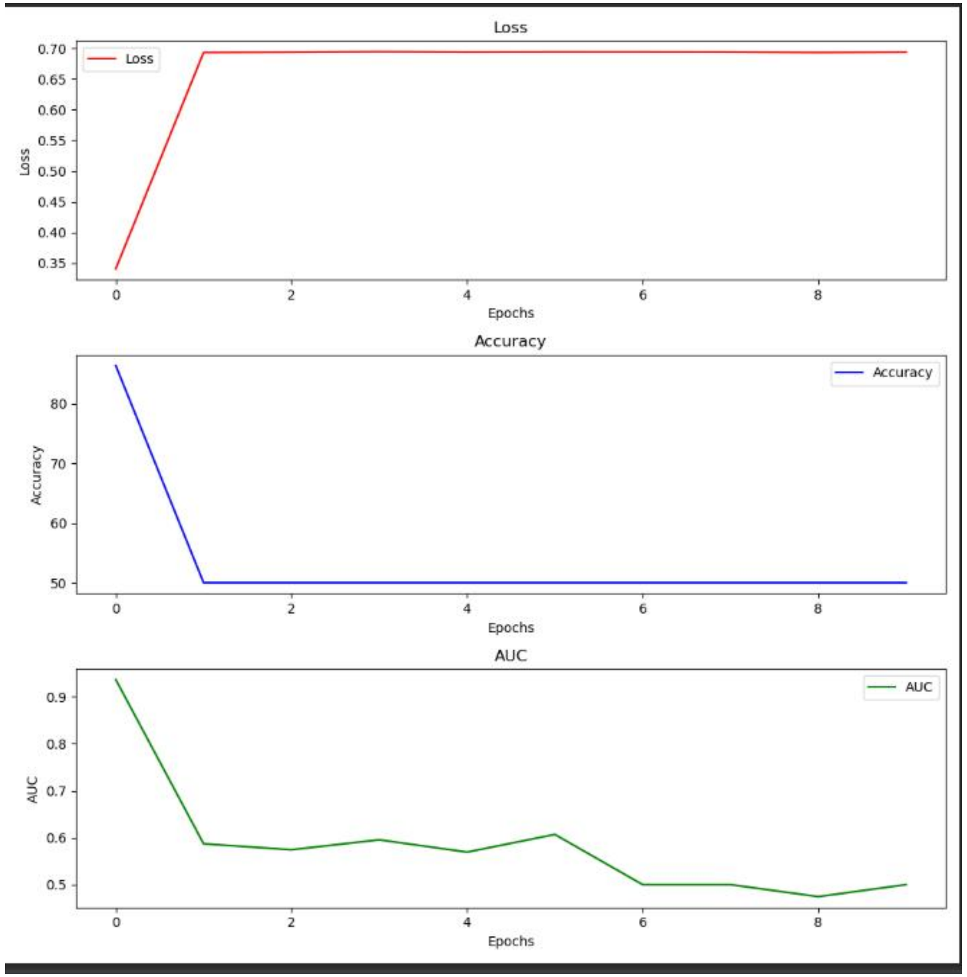

6mA_C.equisetifolia

StableDNAm

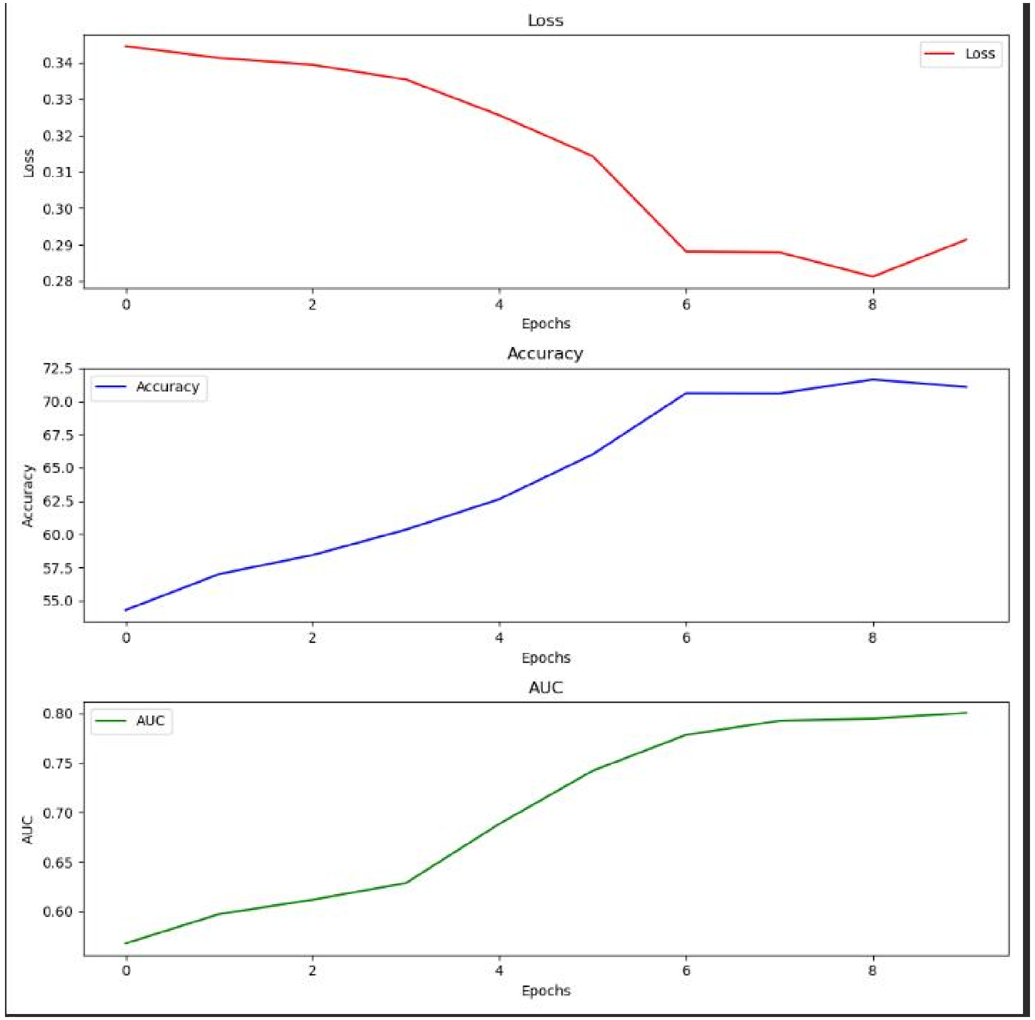

iDNA-ABF

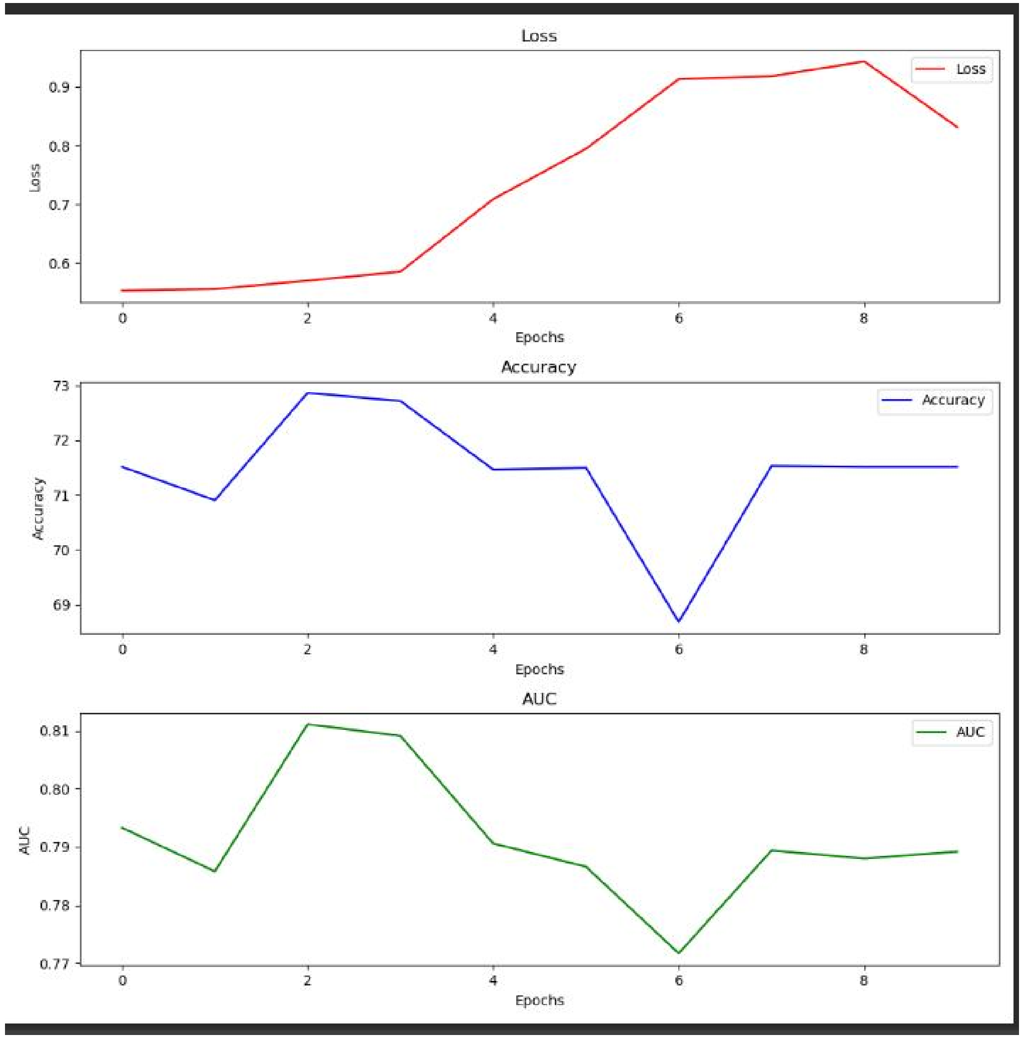

6mA_D.melanogaster

StableDNAm

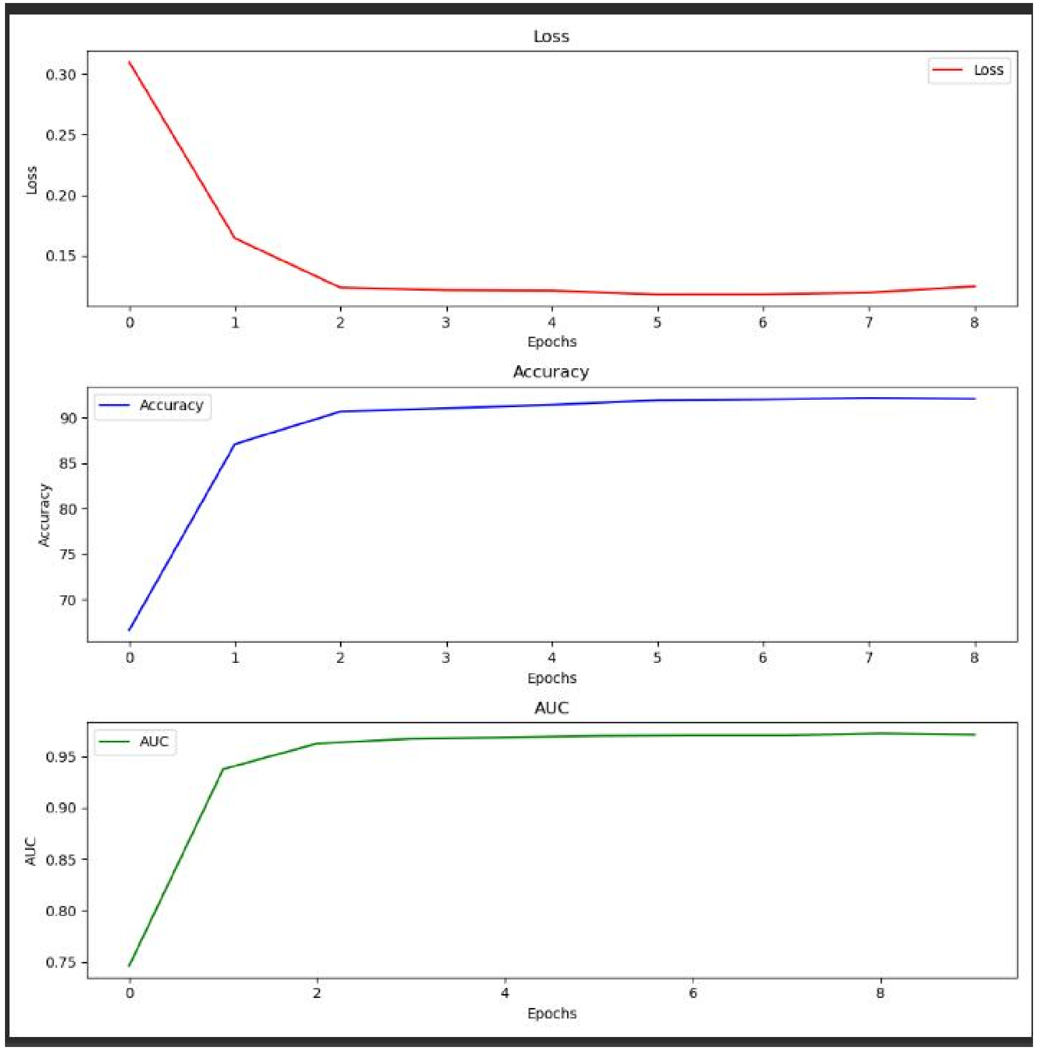

iDNA-ABF

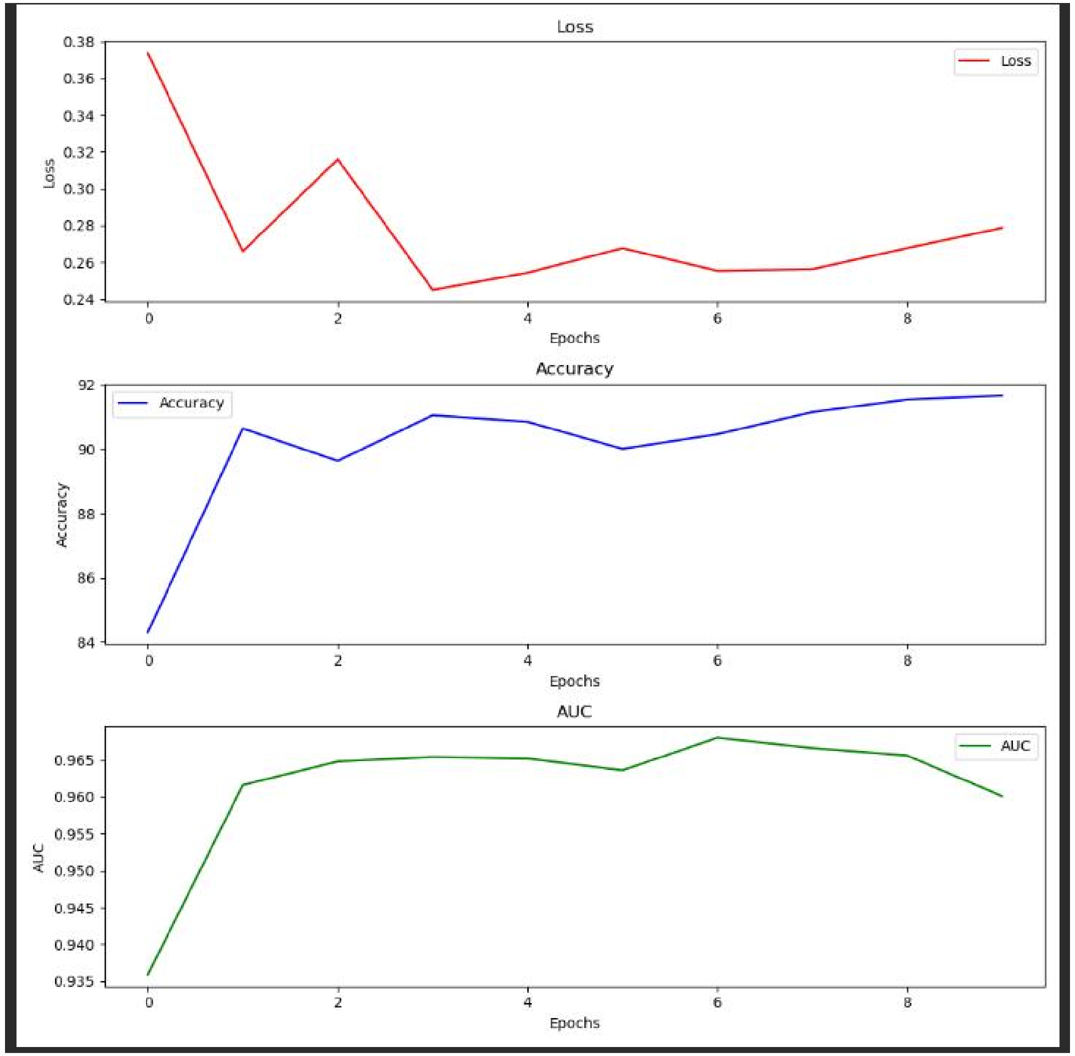

6mA_F.vesca

StableDNAm

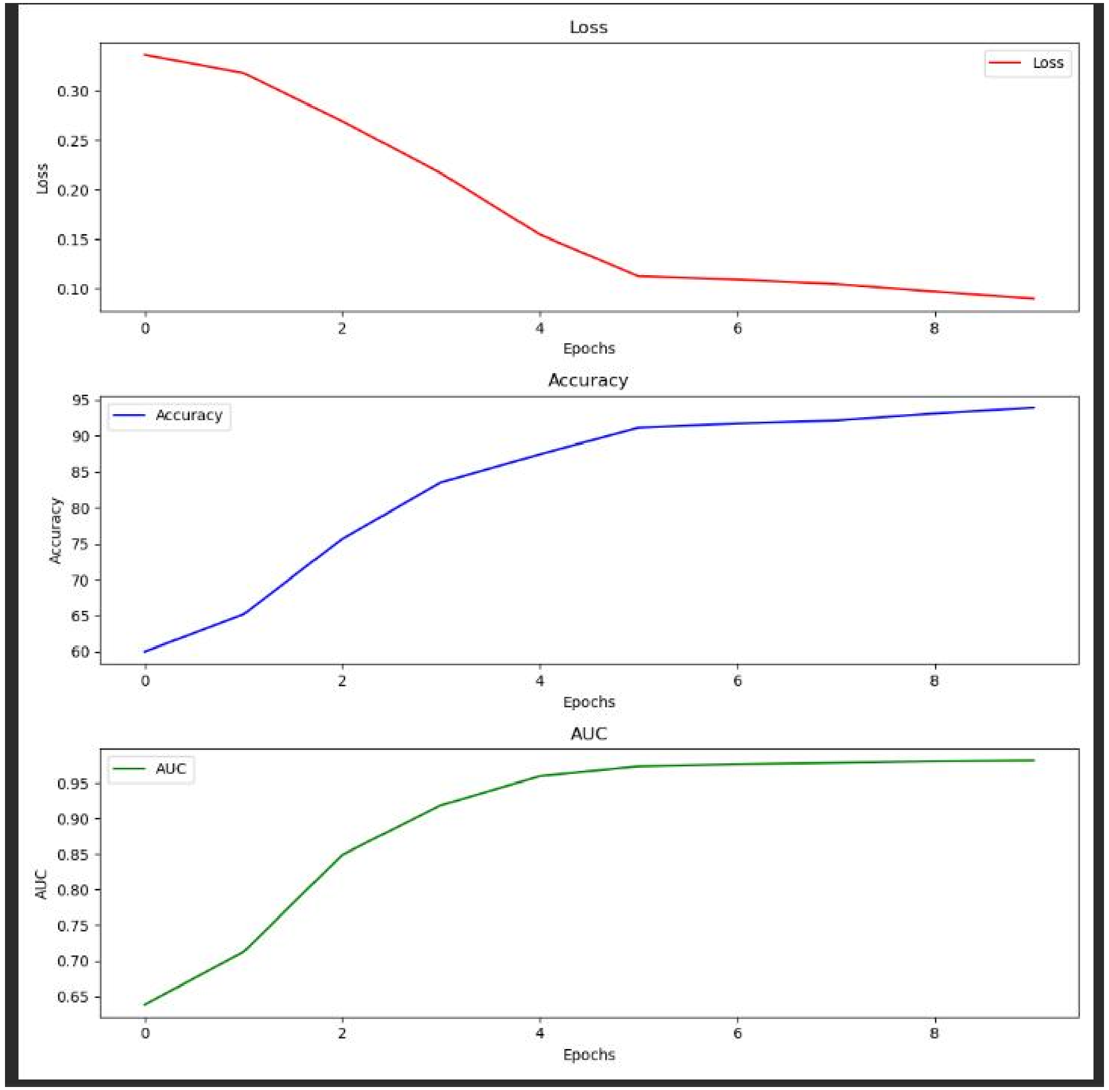

iDNA-ABF

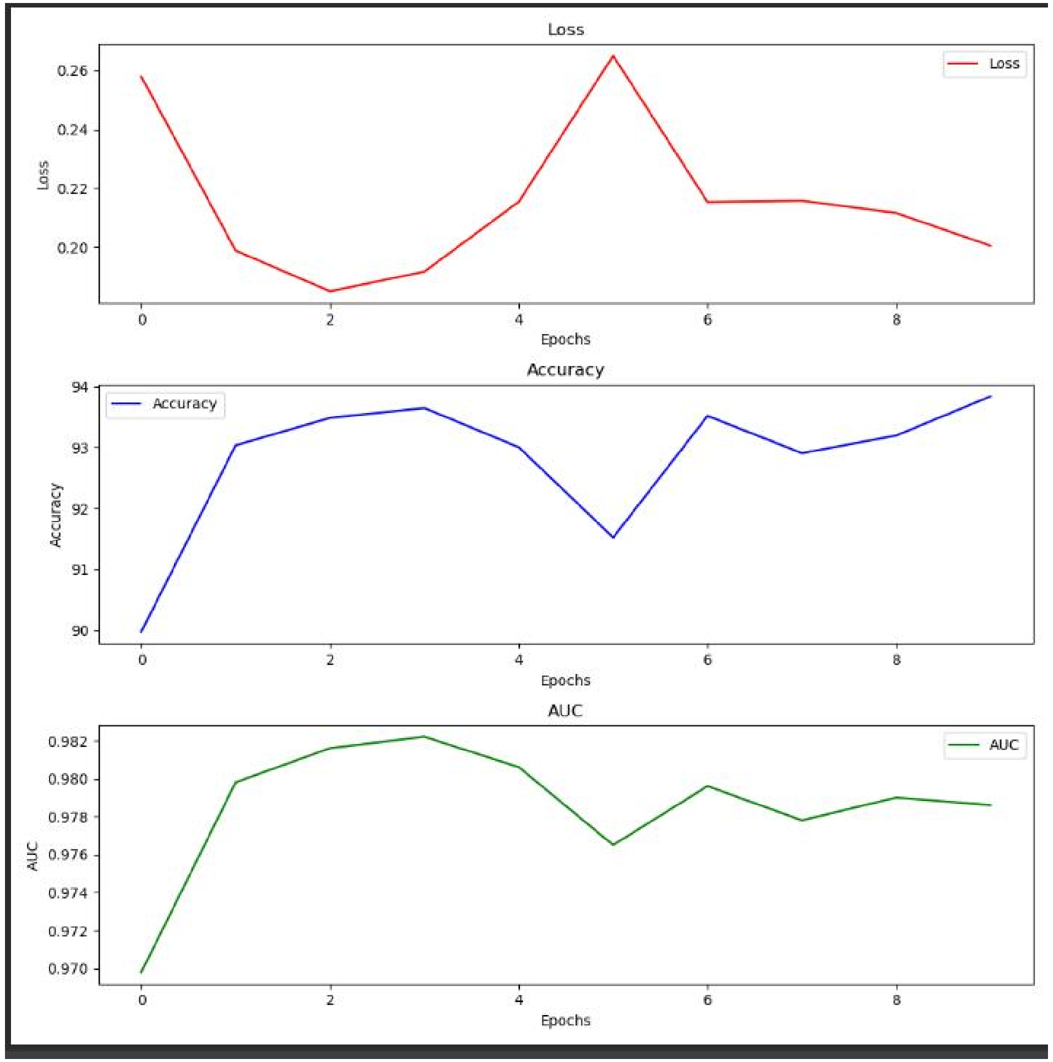

6mA_H.sapiens

StableDNAm

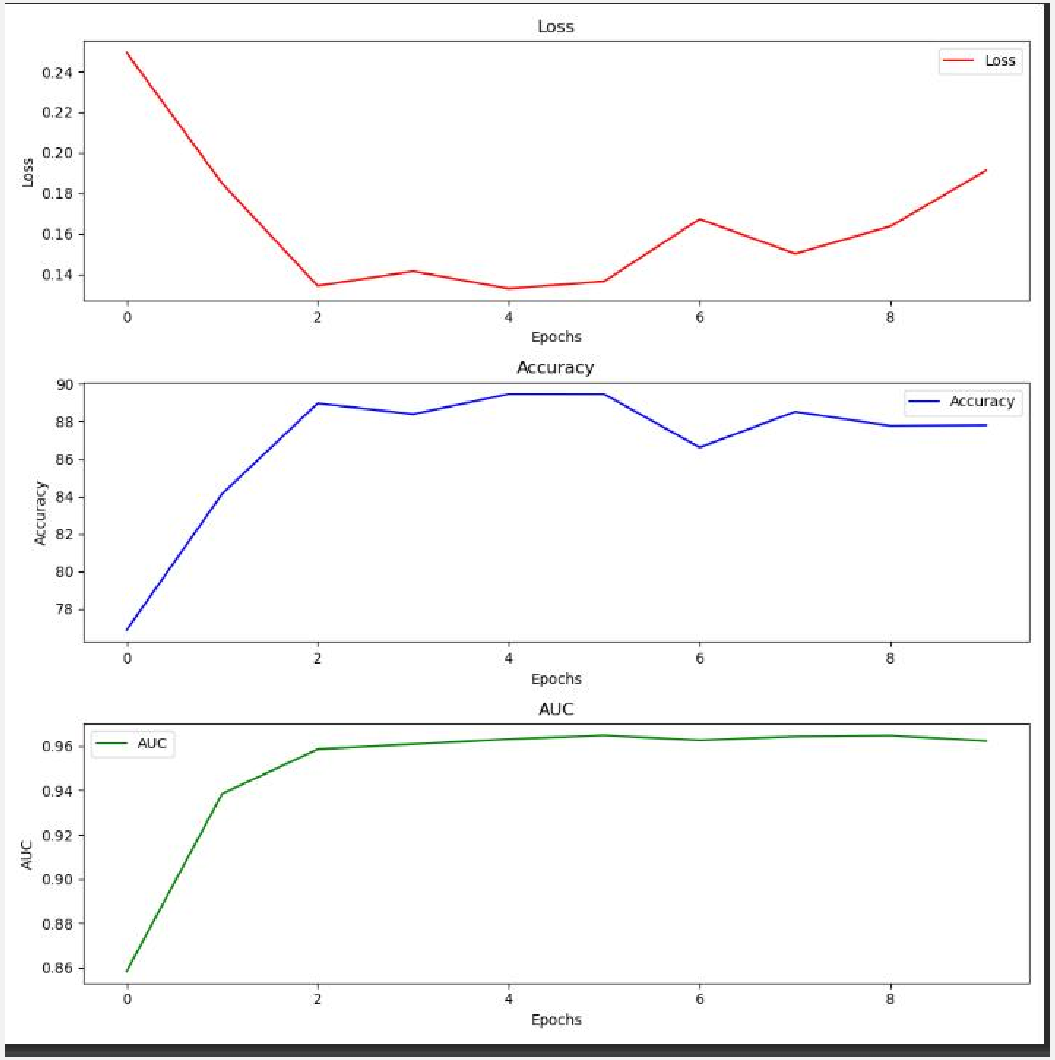

iDNA-ABF

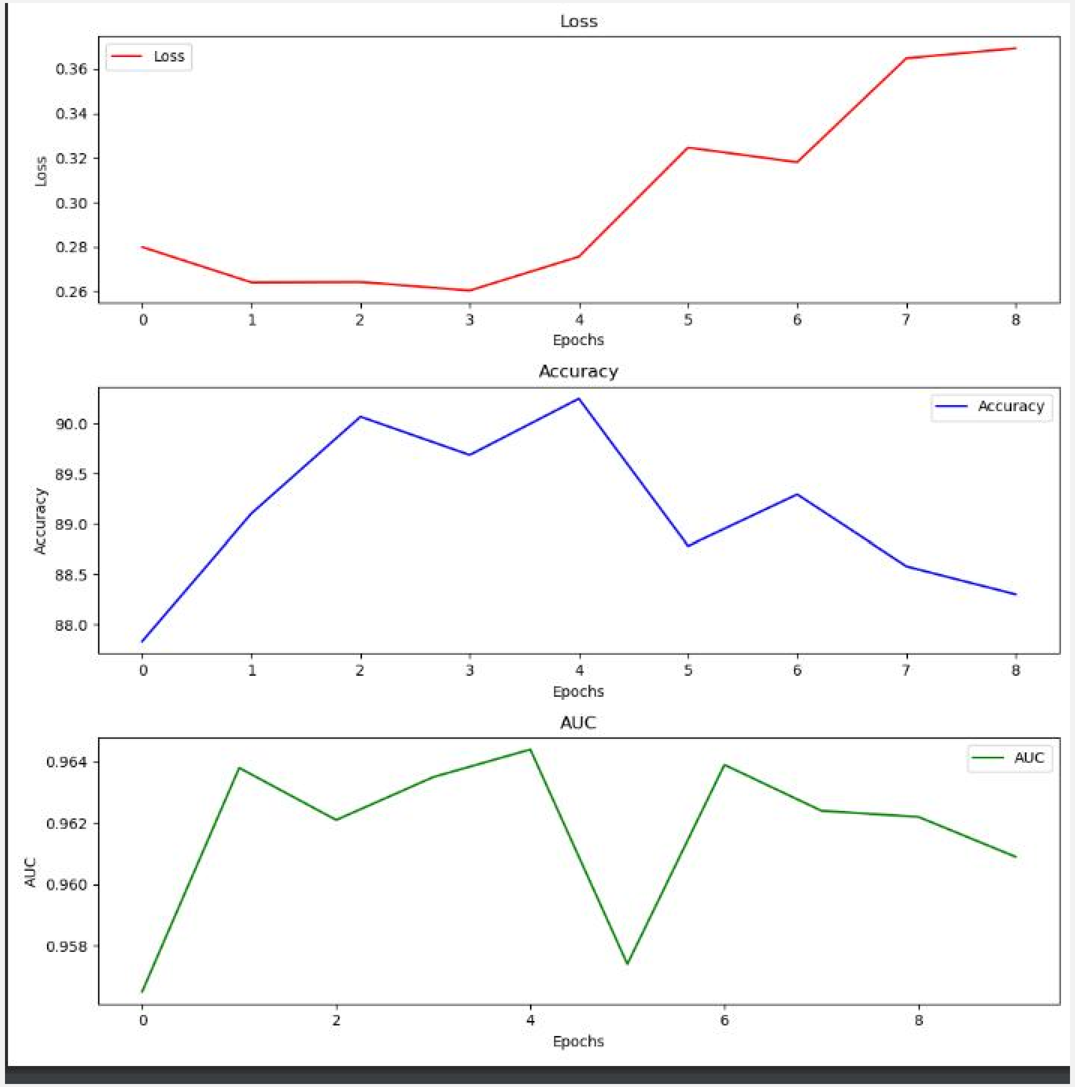

6mA_R.chinensis

StableDNAm

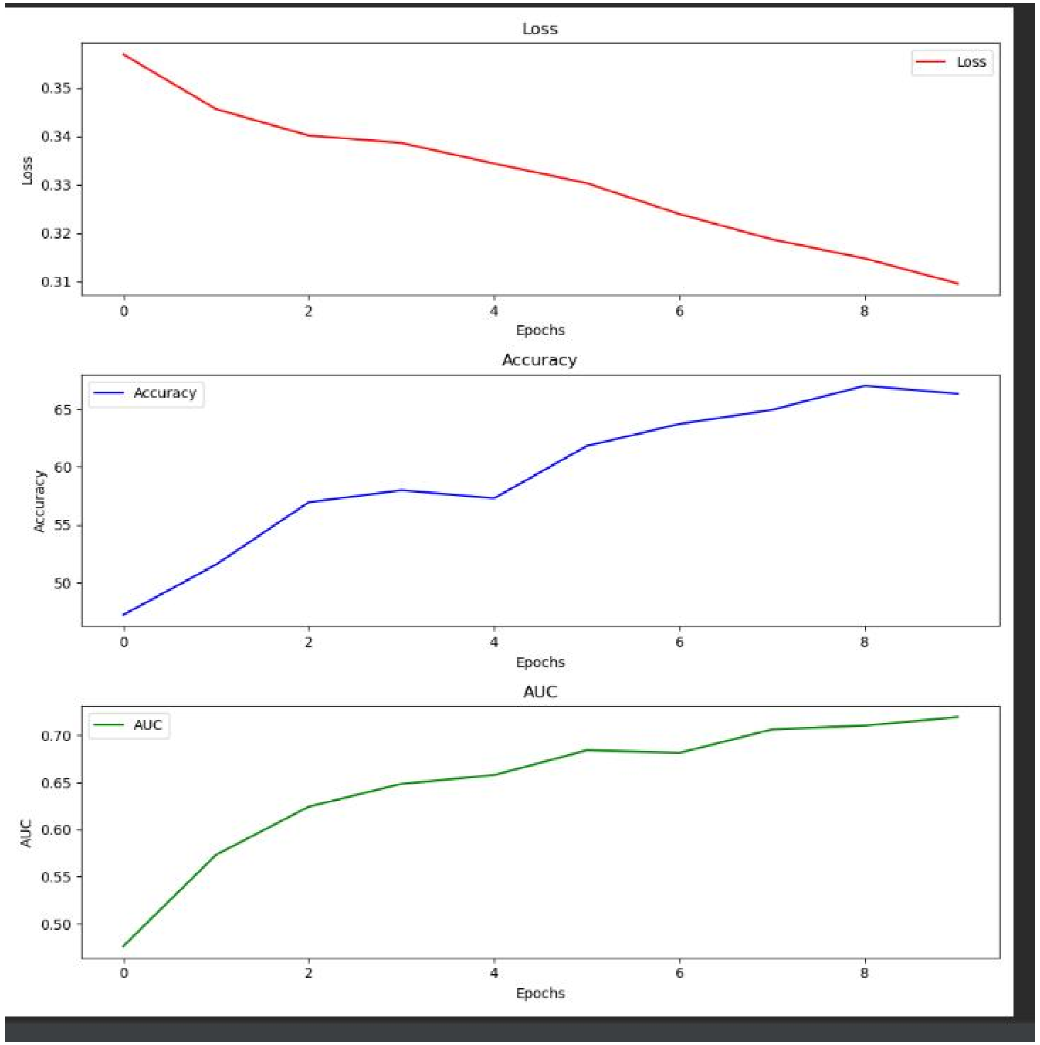

iDNA-ABF

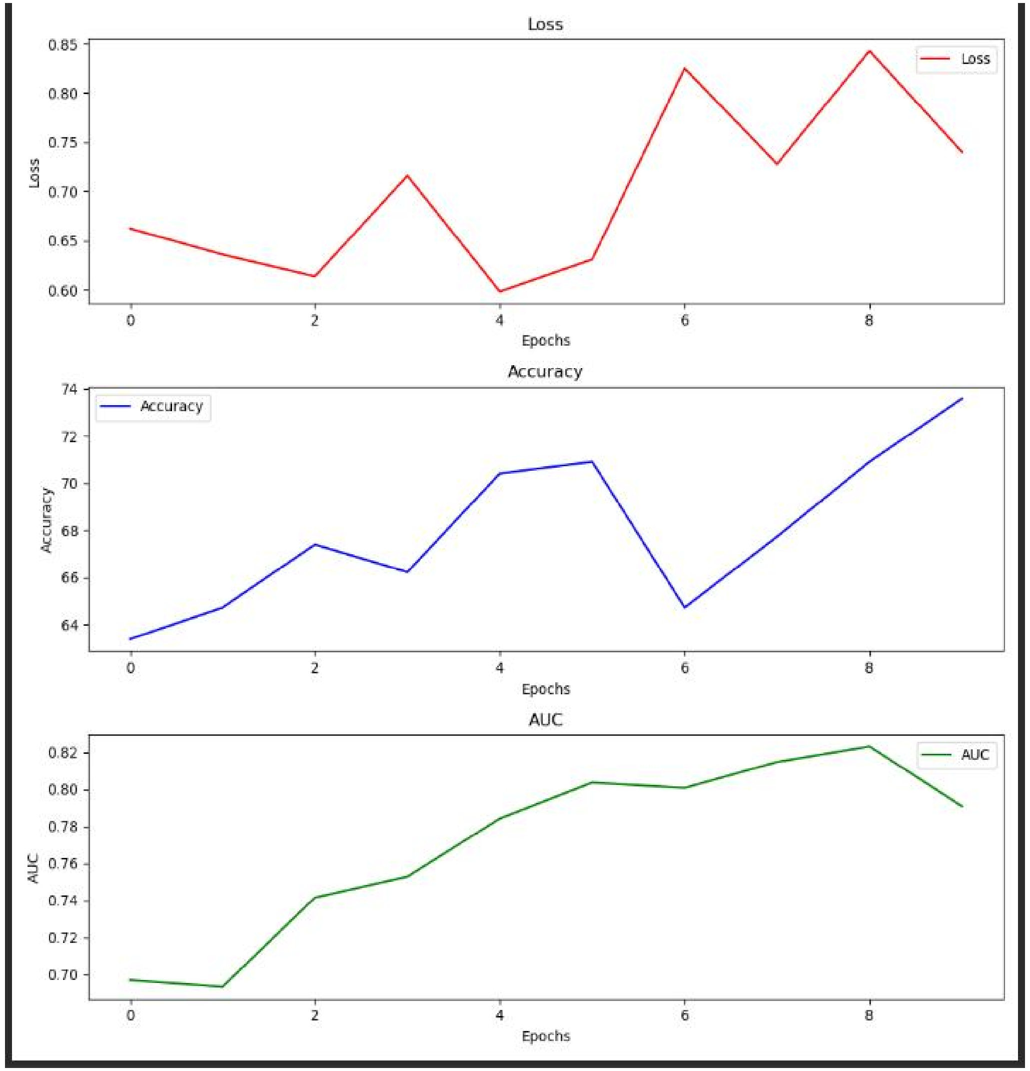

6mA_S.cerevisiae

StableDNAm

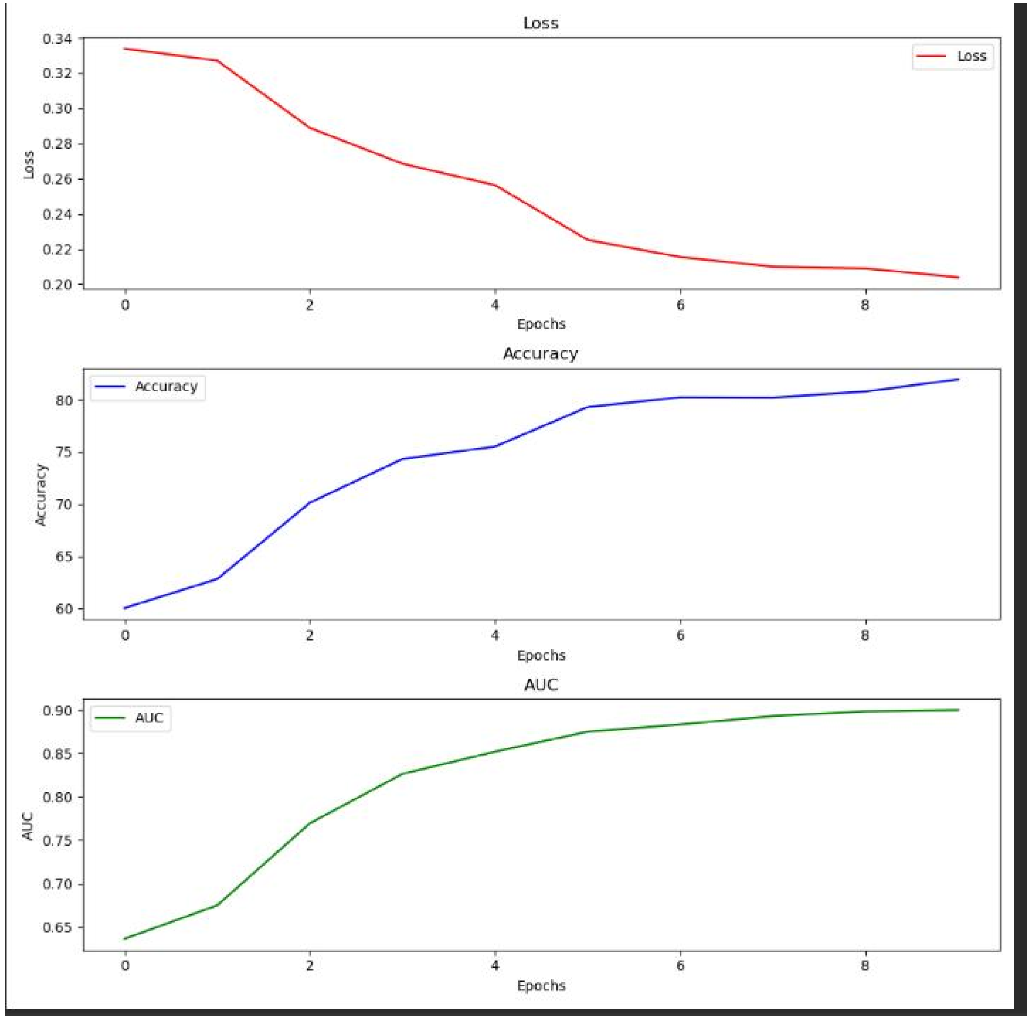

iDNA-ABF

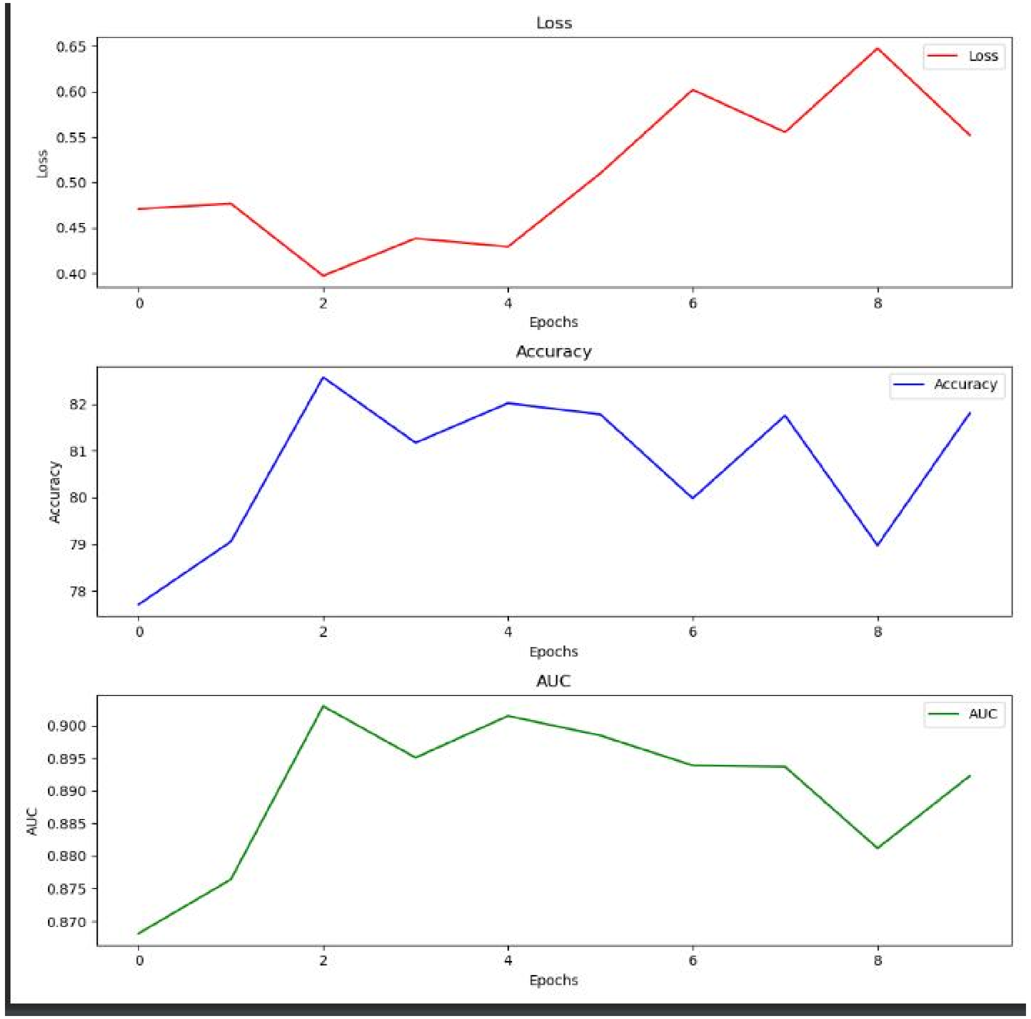

6mA_T.thermophile

StableDNAm

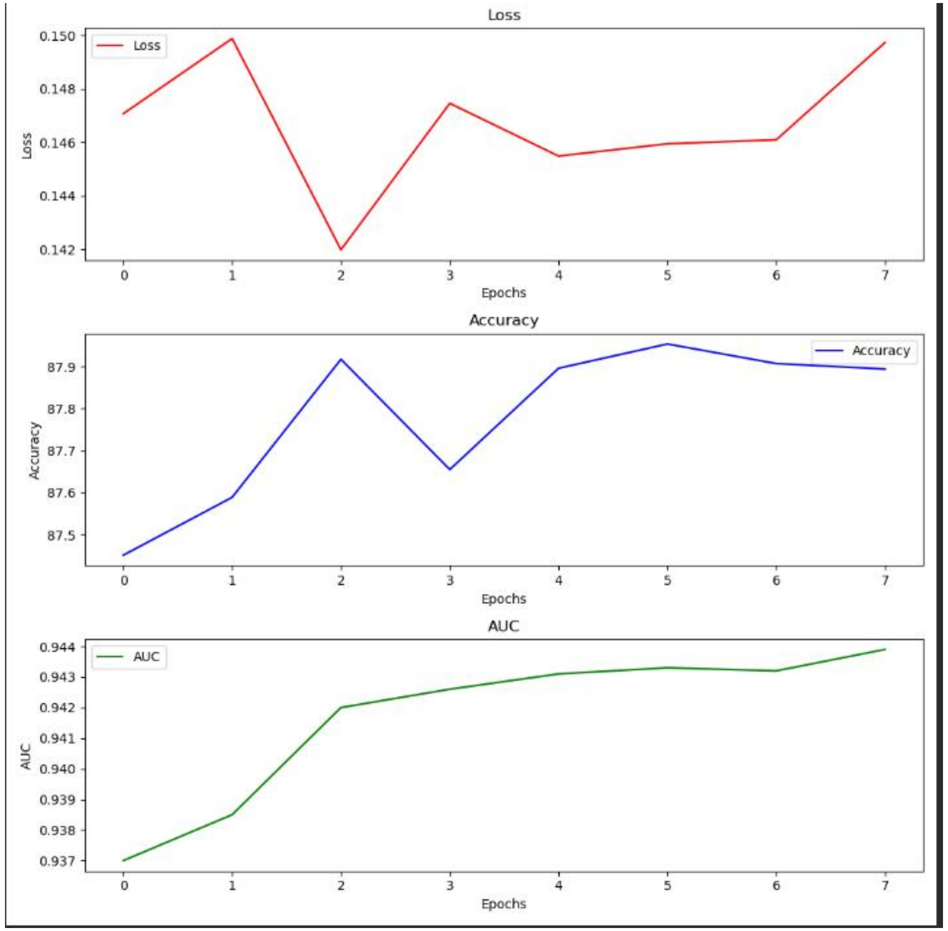

iDNA-ABF

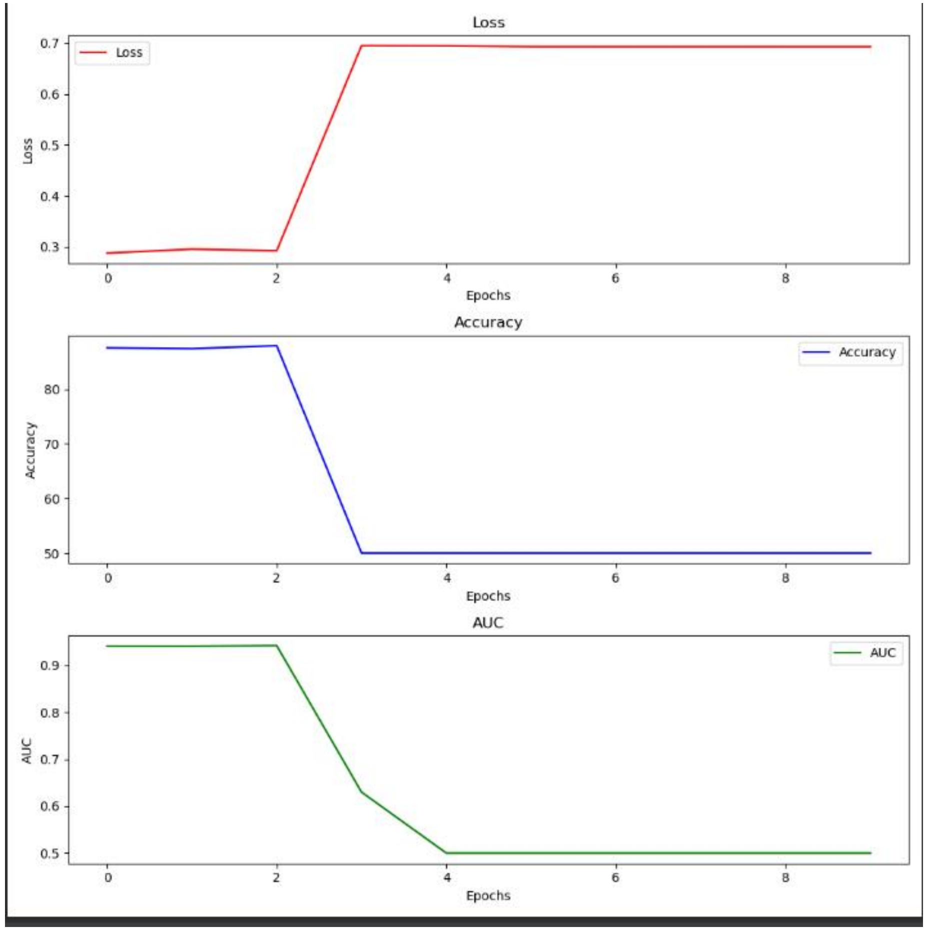

6mA_Tolypocladium

StableDNAm

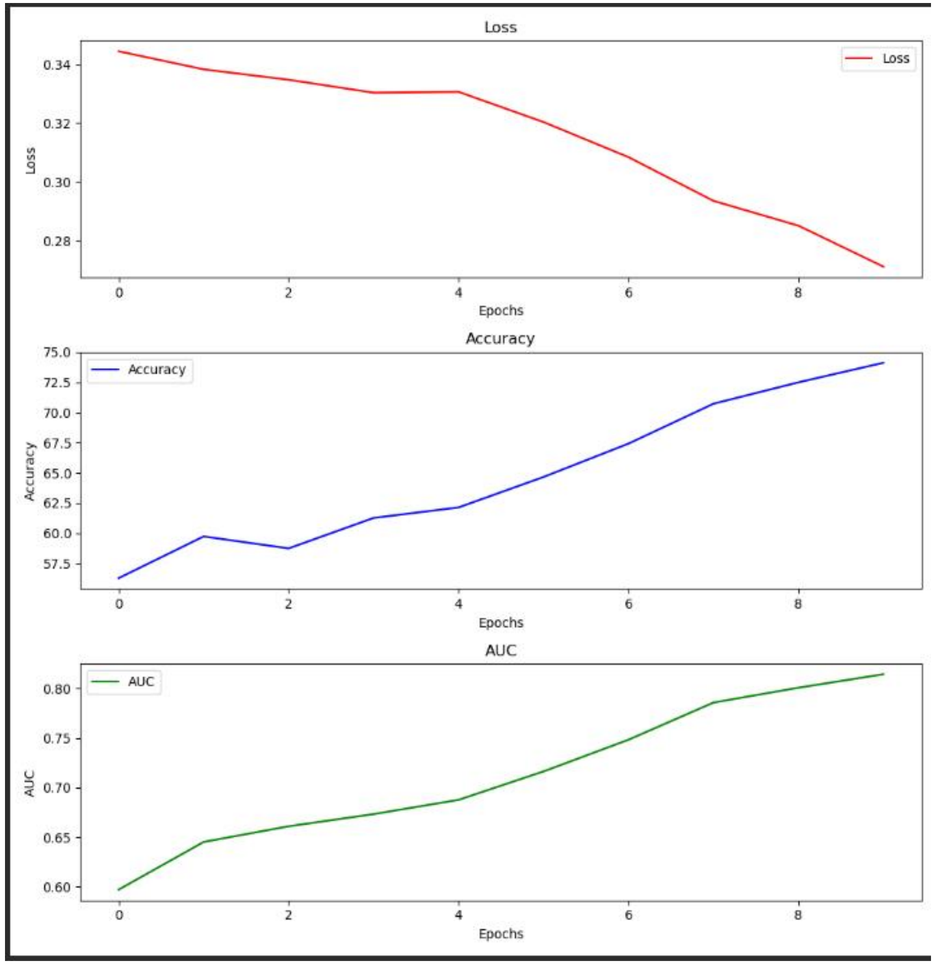

iDNA-ABF

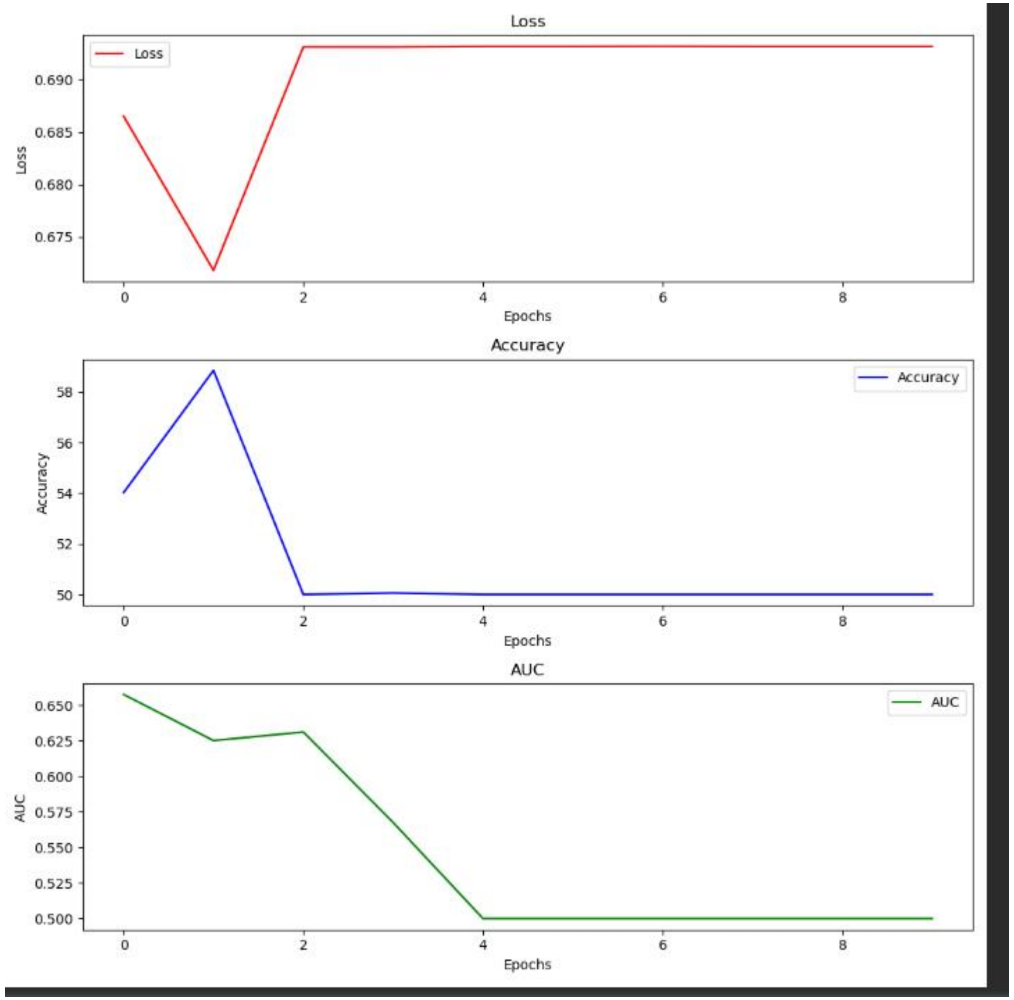

6mA_Xoc BLS256

StableDNAm

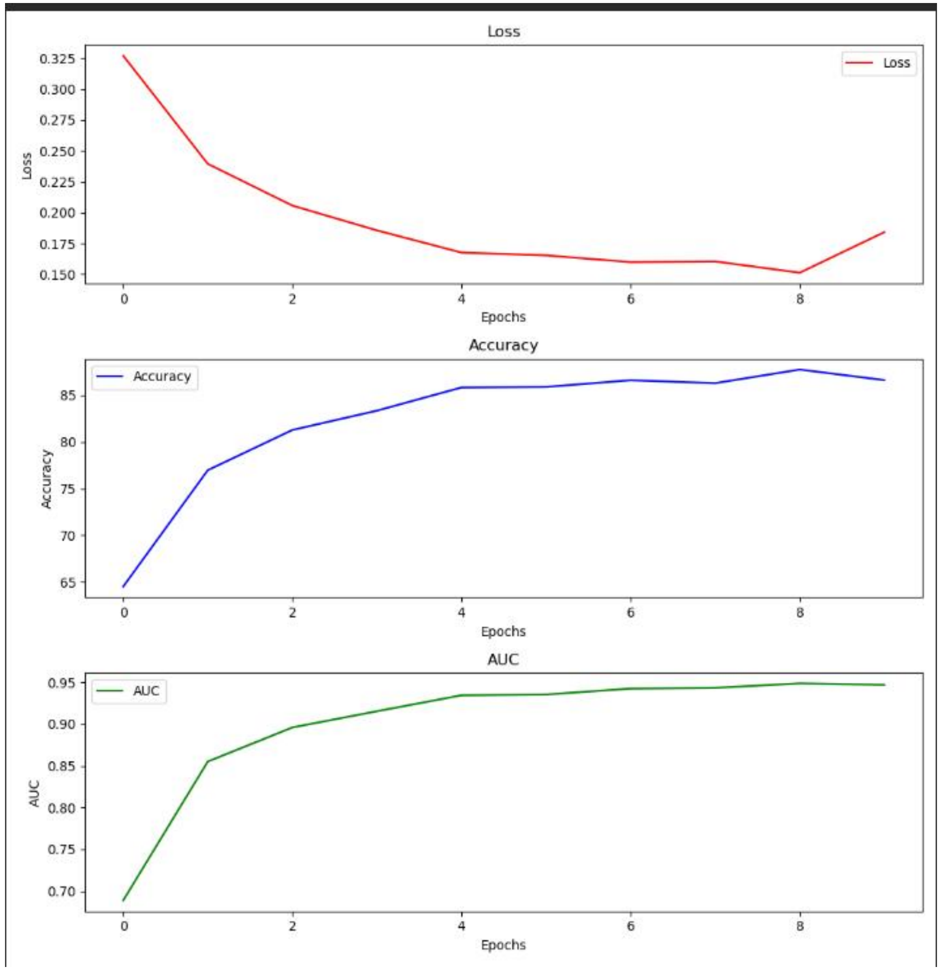

iDNA-ABF

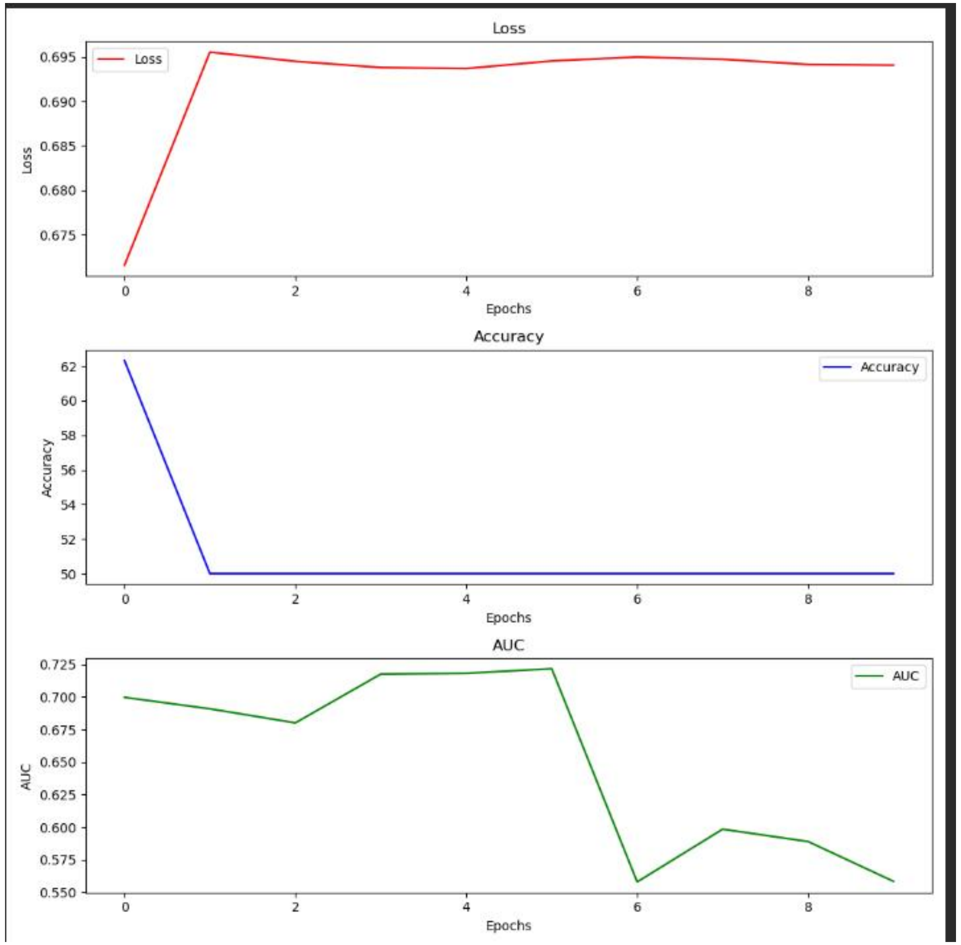

Unified Dataset

StableDNAm

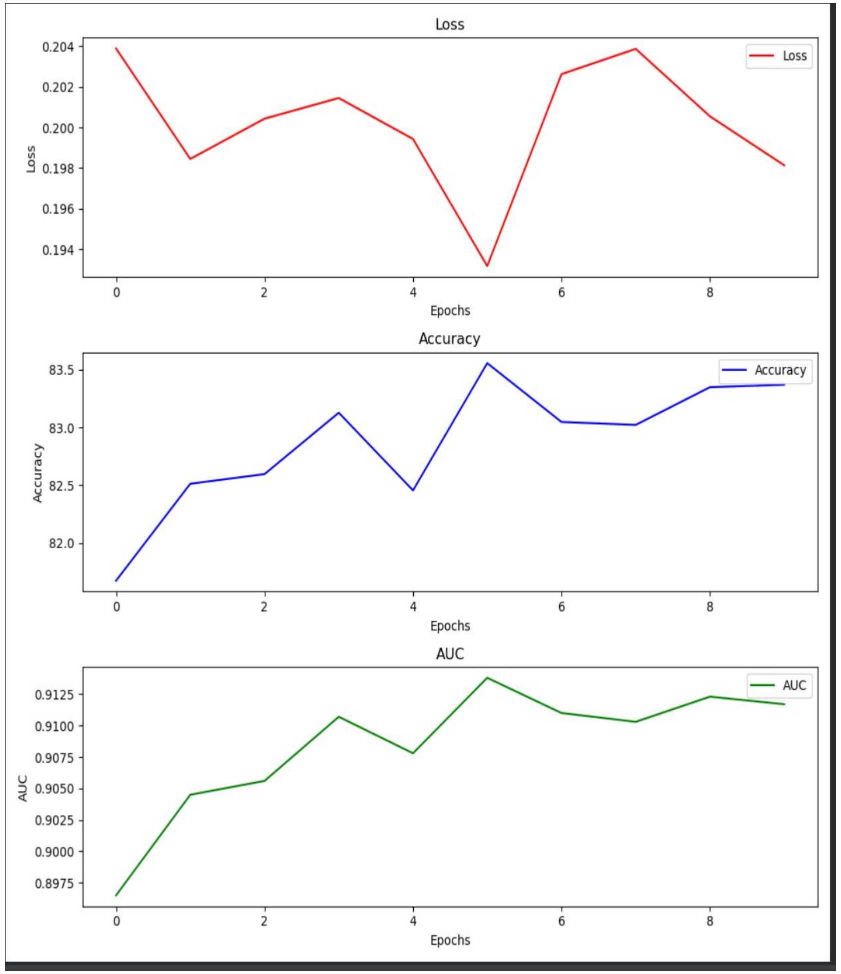

iDNA-ABF

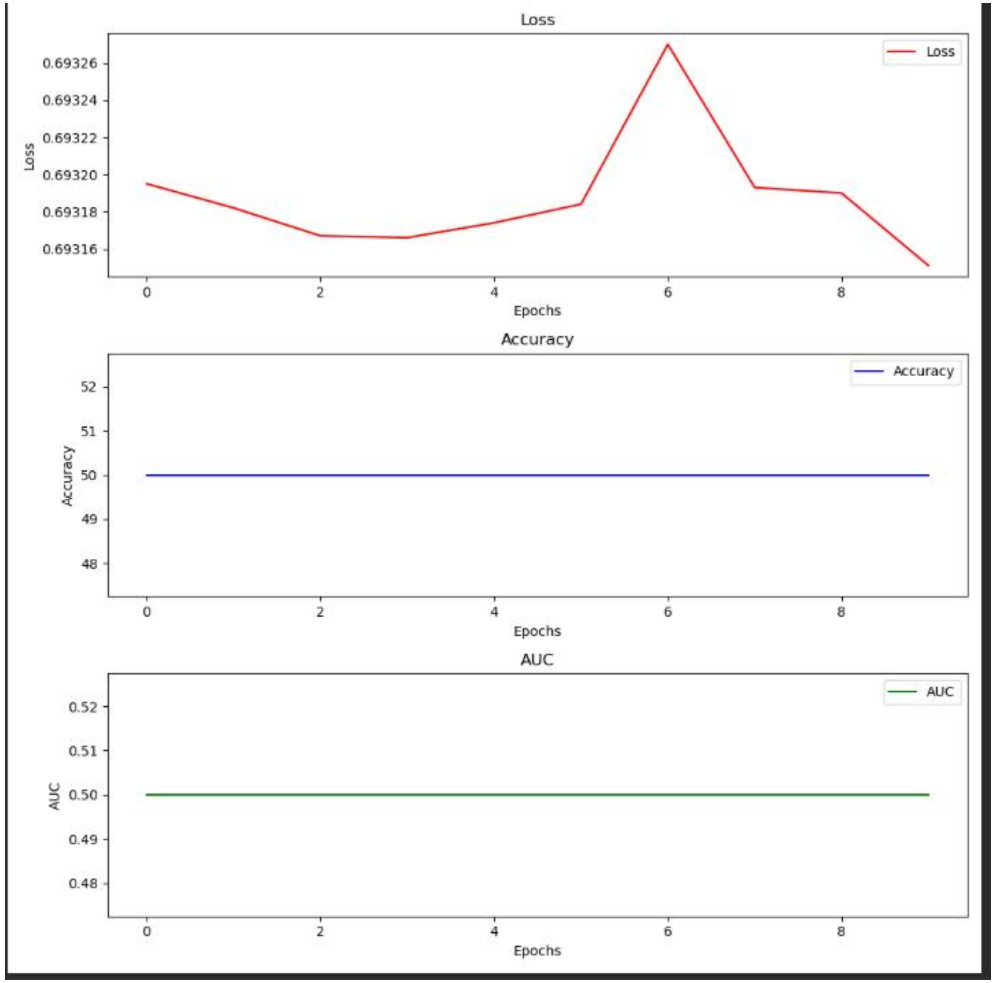

Supplement: Supplementary file 1 — Additional file 1. [file 12864_2023_9802_MOESM1_ESM.zip › Supplementary-Results/All-curves.pdf]
