# Supplementary material for: StableDNAm: towards a stable and efficient model for predicting DNA methylation based on adaptive feature correction learning
Source: BMC Genomics. 2023 Dec 5;24:742. doi: 10.1186/s12864-023-09802-7 (PMC10698904; doi:10.1186/s12864-023-09802-7)
Supplement: Supplementary file 1 — Additional file 1. [file 12864_2023_9802_MOESM1_ESM.zip › Supplementary-Results/training_parameters.pdf]

StableDNAm:

threshold = 0.95
epoch = 30
batch-size = 64
lr =0.00005

iDNA-ABF:

threshold = 0.95
epoch = 20
batch-size = 16
lr = 0.00005

iDNA-ABT:

threshold = 0.7
max-len = 256
num-layer = 3
num-head = 8
dim-embedding = 32
dim-feedforward = 64
dim-k = 3
dim-v = 3
lr = 0.0001
batch-size = 64
epoch = 100

BERT6mA :

threshold = 0.5
batch-size = 128
epoch = 50
lr=0.005
Deep6mA:
lr =0.01
batch-size = 256
epoch = 50

MM-6mAPred :

batch-size =128
lr=0.0001
epoch = 60

iDNA-MS :

Batch size =256

lr=0.0005

epoch = 70

Model	Time	Parameters
StableDNAm	1	11B
iDNA-ABF	0.15	1.7B
iDNA-ABT	0.0002	0.0001B
BERT6mA	0.06	7B
MM-6mAPred	0.001	0.014B
iDNA-MS	0.01	0.15B

Here, "time" refers to the time it takes for one epoch on machine (A40). Typically, it takes around 10 minutes to complete one epoch. Additionally, "parameters" refer to the number of model parameters. Due to differences in device performance, other model run times may vary on different devices.
